# High-intensity interval training ameliorates Alzheimer's disease-like pathology by regulating astrocyte phenotype-associated AQP4 polarization

**DOI:** 10.7150/thno.81951

**Published:** 2023-06-04

**Authors:** Shu Feng, Chongyun Wu, Peibin Zou, Qianting Deng, Zhe Chen, Meng Li, Ling Zhu, Fanghui Li, Timon Cheng-Yi Liu, Rui Duan, Luodan Yang

**Affiliations:** 1 School of Physical Education and Sports Science, South China Normal University, Guangzhou 510006, China.; 2 School of Sport Sciences, Nanjing Normal University, Nanjing 210046, China.

**Keywords:** Alzheimer's disease, High-intensity interval training, Astrocyte AQP4, Glymphatic system, Aβ clearance

## Abstract

**Background:** Alzheimer's disease (AD), one of the most common forms of dementia, is a widely studied neurodegenerative disease characterized by Aβ accumulation and tau hyperphosphorylation. Currently, there is no effective cure available for AD. The astrocyte AQP4 polarized distribution-mediated glymphatic system is essential for Aβ and abnormal tau clearance and is a potential therapeutic target for AD. However, the role of exercise on the AQP4 polarized distribution and the association between the AQP4 polarized distribution and astrocyte phenotype polarization are poorly understood.

**Methods:** Using a streptozotocin (STZ)-induced sporadic AD rat model, we investigated the effects of high-intensity interval training on AD pathologies. The Branes maze task was conducted to measure spatial learning and memory. Immunofluorescence staining of NeuN with TUNEL, Fluoro-Jade C, and relative neuronal damage markers was applied to measure neuronal apoptosis, neurodegeneration, and damage. Sholl analysis was carried out to analyze the morphology of microglia. Line-scan analysis, 3D rendering, and the orthogonal view were applied to analyze the colocalization. Western blot analysis and enzyme-linked immunosorbent assay (ELISA) analysis were conducted to examine AQP4 and Aβ, respectively. An APP/PS1 transgenic AD mice model was used to confirm the key findings.

**Results:** High-intensity interval training (HIIT) alleviates cognitive dysfunction in STZ-induced AD-like rat models and provides neuroprotection against neurodegeneration, neuronal damage, and neuronal loss. Additionally, HIIT improved the drainage of abnormal tau and Aβ from the cortex and hippocampus via the glymphatic system to the kidney. Further mechanistic studies support that the beneficial effects of HIIT on AD might be due, in part, to the polarization of glial cells from a neurotoxic phenotype towards a neuroprotective phenotype. Furthermore, an intriguing finding of our study is that the polarized distribution of AQP4 was strongly correlated with astrocyte phenotype. We found A2 phenotype exhibited more evident AQP4 polarization than the A1 phenotype.

**Conclusion:** Our findings indicate that HIIT ameliorates Alzheimer's disease-like pathology by regulating astrocyte phenotype and astrocyte phenotype-associated AQP4 polarization. These changes promote Aβ and p-tau clearance from the brain tissue through the glymphatic system and the kidney.

## Introduction

Alzheimer's disease (AD), one of the most common types of dementia, accounts for around two-thirds of cases of dementia in individuals over 65 1, 2. AD currently affects more than 6 million individuals in the US and is expected to increase with improved life expectancy 3. The progressive accumulation of β-amyloid (Aβ) and hyperphosphorylated tau-formed neurofibrillary tangles (NFTs) are the two core pathologies, contributing to and exacerbated by mitochondrial dysfunction, oxidative damage and excessive neuroinflammation 4, 5. Ample evidence suggests that the ineffective removal of hyperphosphorylated tau and Aβ contributes to the initiation and progression of AD 6.

The glymphatic system, also known as the perivascular system, was first described in 2013 as a glial-dependent perivascular network that is indispensable for the clearance of waste metabolites and neurotoxic molecules 6, 7. The glymphatic system moves the cerebrospinal fluid (CSF) from the perivascular space along cerebral arteries, where the CSF mixes with the interstitial fluid (ISF) and parenchymal solutes, followed by exiting down perivenous spaces 8. As such, the metabolic waste and neurotoxic peptides/protein, including lactate, Aβ, and abnormal tau, were removed through the glymphatic system 8. The efficiency of the glymphatic system depends on aquaporin channel 4 (AQP4), a membrane-bound water-channel protein 8, 9. AQP4 is mainly expressed in brain astrocytes and epithelial cells in various peripheral organs 6, 10. The AQP4 at the perivascular astrocyte endfood is indispensable for fluid transport in the perivascular glymphatic pathway 6, 11. In a recent study, astrocyte AQP4 is classified into n-AQP4 located in the neuron-facing membrane domain and p-AQP4 located perivascular endfoot membrane 6. The astrocyte n-AQP4 and p-AQP4 are responsible for the drainage of abnormal tau and the overall brain wastes to peripheral tissues, respectively 6. Notably, the reactive astrocytes in AD were polarized from the A2 anti-inflammatory phenotype to the A1 pro-inflammatory phenotype 1, 12. However, the relationship between astrocyte polarization and AQP4 polarized distribution in AD remains unclear. Microglia, another type of glial cell, is essential for neuronal development and innate immune response, including regulating neuronal inflammation and synapse pruning 13, 14. Like astrocytes, activated microglia can also be polarized into M1 pro-inflammatory phenotypes and M2 anti-inflammatory phenotypes 1. The polarization states of microglia and astrocyte are essential in the initiation and progression of AD. However, the relationship between the polarization states of glial cells and the efficiency of the glymphatic system is not yet understood 4.

The currently available treatments for AD have a limited therapeutic effect and cause great controversy 15, 16. Given the limited effects of the current therapies and the high medical costs of medicinal drugs, non-invasive interventions have recently attracted increasing research interest 14, 17. Promisingly, accumulating evidence noted the prophylactic and therapeutic effects of physical exercise 4, 18. For example, the beneficial effects of exercise in AD treatment and prevention include preserving cognitive function, alleviating Aβ accumulation and tau hyperphosphorylation, and alleviating oxidative stress and neuroinflammation 4, 18. While emerging studies, including ours, have demonstrated the beneficial effects of exercise in AD, the underlying mechanisms involved in Aβ and p-tau clearance deserve further investigation. HIIT comprising short bursts of high-intensity exercise training interspersed with low-intensity exercise or rest recovery intervals, has been considered an emerging trend and hot topic in public health promotion 19, 20. Using a streptozotocin (STZ)-induced Alzheimer's disease-like rat model, we conducted the current study to determine the effects of HIIT on AD pathologies and study the relationship between astrocyte polarization and AQP4 polarized distribution in AD.

## Methods

### Animals and experimental design

**Figure 1:**
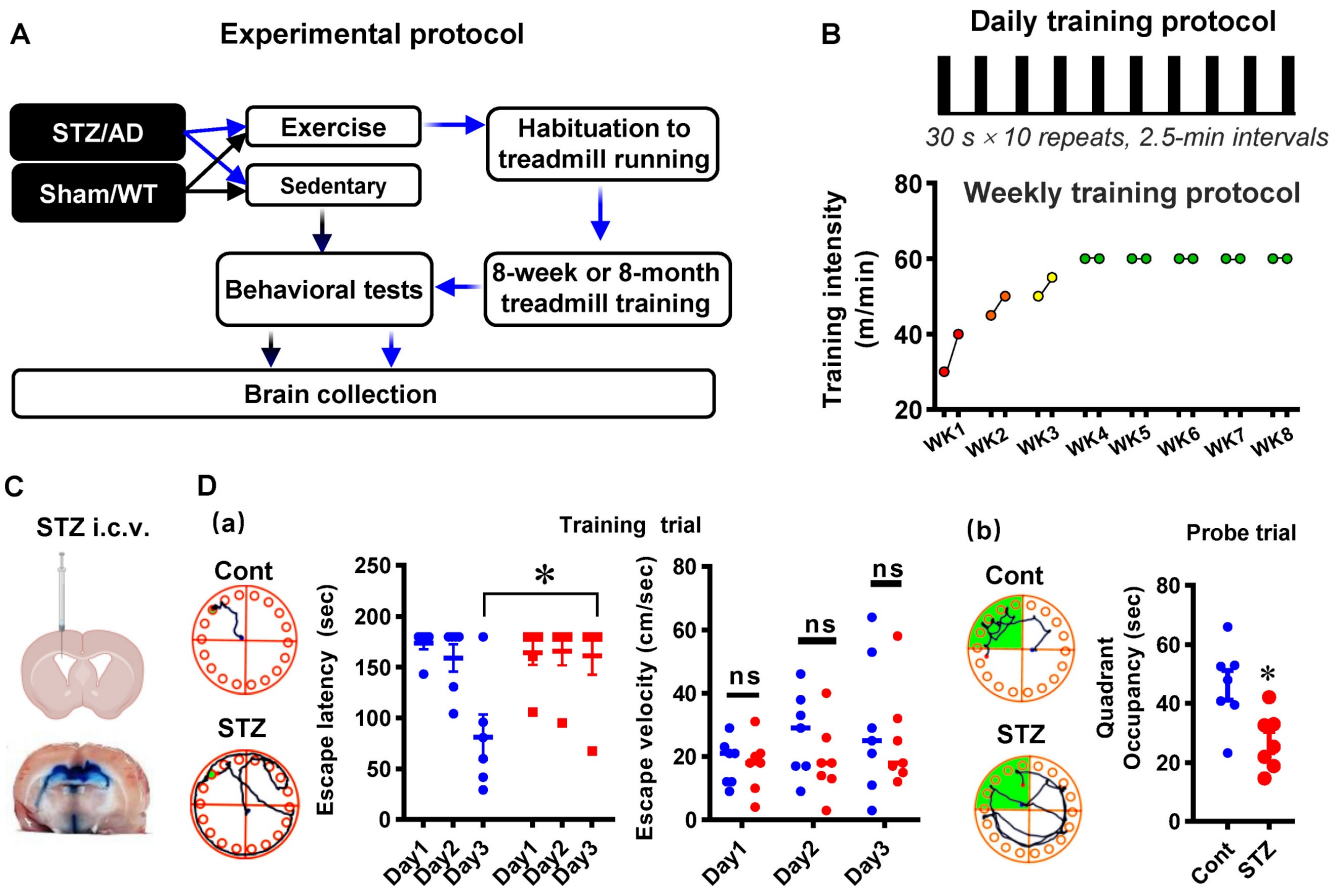
** Diagram of the experimental design and high-intensity interval training protocol. (A)** The control and STZ-injected rats were each randomized into sedentary and exercise groups. Behavioral tests were performed following an 8-week HIIT treatment. Brain collections were initiated after the behavioral test. **(B)** The 8-week HIIT training was performed at 30-40 m/min in the first week, 45-50 m/min in the second week, 50-55 m/min in the third week, and 60 m/min from the fourth to the eighth week. The 30-minute HIIT was carried out 5 days/week with 10 bouts of 30-s sprinting and 2.5 min intervals between each episode. For the transgenic AD mouse model, the 6-month exercise training was initiated at 2 months old and ended at 8 months old with a similar habituation period and training stage. **(C)** Schematic diagram depicting STZ injection into the lateral cerebral ventricle of rats. **(D)** Barnes Maze tests in the established STZ rat model. Data represent mean ± SEM, n = 7. ^*^P < 0.05 vs. Cont group. ns, no significant difference.

Seven-week-old male Sprague-Dawley (SD) rats and the APP/PS1 transgenic AD mice model (Guangdong Medical Laboratory Animal Center) were given one week to adapt to the new environment before experiments. The SD animals were then randomly allocated to the following four groups: 1) Sedentary control group (Cont); 2) Control animals subjected to HIIT (Cont + Exe); 3) Sedentary animals with STZ injection (STZ), and 4) STZ injection subjected to HIIT (STZ + Exe) (**Fig. 1A**). Similarly, the APP/PS1 AD animals were randomly divided into the following groups: 1) Wild type animals (WT); 2) WT+Exe; 3) AD, and 4) AD+Exe (**Fig. 1A**). The HIIT training was performed following a previous study 21. In brief, the training was divided into habituation and HIIT training stages 21. During the 5-day habituation period, the treadmill with no incline was initiated from 5 m/min to 25 m/min for 30 min/day, followed by the HIIT training stage. During the HIIT training stage, as shown in **Fig. 1B**, for the STZ-induced AD animal, the HIIT was performed at 30-40 m/min in the first week, 45-50 m/min in the second week, 50-55 m/min in the third week, and final 60 m/min from the fourth to the eighth week. The 30-minute HIIT was performed 5 days/week with 10 bouts of 30-s sprinting and 2.5 min intervals between each episode. The sedentary animals were also placed on the treadmill with the machine turned off. The STZ-induced AD model underwent 8-week exercise training. For the transgenic AD mouse model, the 6-month exercise training was initiated at 2 months old and ended at 8 months old. All rats were housed in a central animal facility with a 12-h light/dark cycle under ad-lib feeding conditions. All animal care and animal-related experimental procedures were approved by the Institutional Animal Care and Use Committee (IACUC) of South China Normal University and comply with National Institutes of Health guidelines.

### Intracerebroventricular STZ-induced AD model and CSF tracer diffusion experiment

As described in our previous study 22, intracerebroventricular (i.c.v.) streptozotocin (STZ) injection was performed to induce the sporadic Alzheimer's rat model. Briefly, the 30 μg/μl STZ (3 mg/kg) was bilaterally i.c.v injected at a rate of 0.5 μl/min under anesthesia. The procedures were performed using a stereotaxic frame at the following injection coordinates relative to the bregma: lateral ± 1.5 mm, depth -3.8 mm, posterior: -0.8 mm. The control animal underwent identical procedures except injecting with saline rather than STZ. As shown in **Fig. 1C** and **Fig. 1D**, i.c.v injection of Evans blue and behavioral tests were carried out to confirm the successful establishment of animal models. Moreover, the CSF tracer diffusion experiment was performed to analyze the effects of HIIT training on glymphatic transport. Similarly, 8-µl fluorescently labeled Aβ (1 µg/µl, TAMR-Aβ peptide) was bilaterally i.c.v injected following 8-week treadmill training in the STZ-induced AD experiment. The brain and the kidney were collected for further study.

### Barnes Maze Task

As described previously, the Barnes maze task was applied to measure hippocampus-dependent spatial learning and memory 1, 23. Briefly, the Barnes maze task was divided into training days and a probe test. During the training trials, the animals were placed at the center of a circular platform with 18 holes and given 3 min to explore the venue freely. The time spent finding the black escape box (20 ×15 × 12 cm) hidden under one of the holes was recorded during the training trials. If the animals could not find the escape box within 3 min during the training trials, the researcher guided the rats to the black escape box. The escape box was removed during the final 90-s probe test, and the escape hole was blocked. The time spent in the target quadrant and the number of exploring errors were recorded and analyzed by ANY-maze video tracking software (Stoelting, Wood Dale, IL, USA).

### Brain Collection and Tissue Preparation

As shown in **Fig. 1A**, the brain collection was conducted as described in our previous study following the behavioral tests 1, 24. Briefly, the animals were sacrificed under deep anesthesia, followed by transcardial perfusion with ice-cold saline. The whole brain was excised and micro-dissected to get the cortex and hippocampus from half of the brain, followed by post-fixation of the remaining hemisphere. The post-fixed hemisphere was cryoprotected with 30% sucrose and then embedded in the OCT medium, followed by the preparation of the frozen sections (25 μm each) using the Leica Rm2155 microtome. The micro-dissected cortex and hippocampus were frozen in liquid nitrogen and homogenized for future use. As previously described, the frozen tissues were homogenized in a mixture of homogenization medium, protease, and phosphatase inhibitors (Thermo Scientific, Rockford, IL) 22, 25. The protein concentrations were determined using Modified Lowry Protein Assay (Pierce, Rockford, IL).

### Immunofluorescence Staining

The cortex and CA1 area of the hippocampus were chosen for immunofluorescence staining and analysis 1, 26. Briefly, the coronal brain sections were permeabilized for 3 x 20 min with 0.4% Triton X-100 (Thermo Fisher), followed by 60 min blocking with 10% normal donkey serum. The brain slices were then incubated at 4 °C with the following primary antibody overnight: anti-NeuN (Abcam), Bax (HA), Bcl-2 (Arigo), Cle-caspase 3 (Proteintech), Cle-caspase 9 (HUABIO), MAP2 (Abcam), MBP (Abcam), synaptophysin (Abcam), spinophilin (Abcam), Iba-1 (FUJIFILM Wako), CD86 (Abcam), CD206 (Proteintech), GFAP (Abcam), C3d (Bio-Techne), S100A10 (Thermo Fisher), RECA1 (Bio-Rad), AQP4 (Abcam), Aβ1-42 (Cell Signaling), and PHF1 (AB Clonal). Afterward, the brain slices were washed 3 x 10 min with 0.1% Triton X-100 and incubated with appropriate secondary antibodies (Alexa Fluor 488/594/647-nm donkey anti-mouse/rabbit/goat IgG) for 1 h at room temperature. After 5 x 10 min with 0.1% Triton X-100-PBS, the brain slices were coverslipped DAPI Fluoromount-G® Mounting Medium (SouthernBiotech). The confocal images were captured on an LSM800 confocal laser scanning microscope. The images were viewed and analyzed using Fiji-ImageJ software (Version 2.9.0, NIH, Bethesda, MD, USA) or Imaris software (Version 9.7.0, Bitplane AG, Zürich, Switzerland). Line-scan analysis of the representative images was performed using Fiji-ImageJ and created by GraphPad Prism 8 software. Microglial Sholl analysis and 3D rendering were performed using Fiji-ImageJ software and Imaris software. The smoothing and threshold adopted in 3D rendering were set following our previous study 1. Furthermore, as mentioned previously, n-AQP4 is the astrocyte AQP4 located in the neuron-facing membrane domain, and the p-AQP4 is the astrocyte AQP4 located in the perivascular endfoot membrane, according to the previous study 6. Image J software with MorphoLibJ plugin and ROI Manager to measure and qualify the AQP4 intensity and colocalization. The intensity of AQP4 co-colocalized with RECA1 was qualified as p-AQP4, and the intensity of AQP4 co-colocalized with NeuN was qualified as n-AQP4.

### Fluoro-Jade C Staining and TUNEL assay

As previously described, the Fluro-jade C staining was conducted to measure neuronal degeneration 27. Briefly, we incubated the brain slices in the dark with the Fluoro-Jade C working solution (Sigma-Aldrich, St. Louis, MO, USA) dissolved in 0.1% acetic acid for 20 min at room temperature. After three washes with distilled water, the brain slices were coverslipped for confocal microscopy with a mounting medium. Double staining of the brain slices with the NeuN and a Click-iT® Plus TUNEL assay kit (Thermo Fisher Scientific) were adopted to mark the apoptotic neurons 4, 27. The TUNEL was performed following the manufacturer's instructions and our previous study 1.

### Western Blotting Analysis

Western blotting was carried out as we previously described 22. The extracted proteins were boiled with a loading buffer at 100 °C for 5 min, then separated on 12% sodium dodecyl sulfate-polyacrylamide gel (SDS, 30 μg per lane) 28 and transferred onto polyvinylidene difluoride (PVDF) membrane. The protein-loaded membrane was then blocked with 3% bovine serum albumin (BSA), followed by incubation with AQP4 (Abcam) and β-actin (Beyotime) overnight. After three washes with 0.2% tween-20, the membrane was incubated with horseradish peroxidase (HRP)-conjugated secondary antibodies (Cell Signaling) at room temperature for 1 h. Subsequently, a CCD digital imaging system (ImageQuant LAS 4000, GE Healthcare) was applied to view and capture the images after adding the HRP chemiluminescent substrate. The images of bound proteins were analyzed with Image J software (NIH, USA), and the results of band densities were normalized to that of β-actin.

### Hematoxylin and Eosin (H&E) staining

The kidney tissue was removed quickly and embedded in tragacanth (Millipore Sigma). The frozen sections in 5 μm thickness were prepared using the Leica Rm2155 microtome, followed by staining with HE histopathological examination following the manufacturer's instructions.

### Measurement of Aβ and p-tau levels

A rat Aβ1-42 ELISA kit (Jianglai, JL10958) was used to assess the levels of Aβ1-42 in the brain and kidney tissue following the manufacturer's instructions. In brief, equal amounts of protein samples (20 μg) from the brain and kidney tissue were incubated in the pre-coated microplate for 1 h at 37 °C. After 3 rinses, the microplate was incubated with 100-μl detection antibody for another 1 h at room temperature, followed by incubation with HRP-conjugated secondary at 37 °C for 15 min. The optical density was finally read at 450 nm using a microplate reader (Bio-Rad). In addition, a rat phosphor-tau ELISA (Tzybiotech, TK05791) was used to measure the levels of p-tau in brain and kidney tissues. Similarly, 100 μl of samples with equal amounts of protein were incubated within the pre-coated microplate for 1 h at 37 °C, followed by incubation with manufacturer-provided solution A for another hour at 37 °C. After 3 washes with distilled water, manufacturer-provided solution B was added and incubated for 30 min. Finally, after adding the TMB (3,3′,5,5-tetramethylbenzidine) chromogen solution and stop solution, the microplate was read at 450 nm. All data were calculated and presented as the percentage of the STZ group.

### Statistical analysis

The SigmaStat (Systat Software; San Jose, CA, USA) software was adopted to perform statistical analysis. One-way analysis of variances (ANOVAs) or two-way ANOVA with Student-Newman-Keuls (S-N-K) post hoc tests were conducted to determine group differences. When only two groups were included in the statistical analysis, a Student's *T* test was performed. All data were expressed as mean ± standard error (SE). A p-value less than 0.05 indicated a significant difference between groups.

## Results

### HIIT ameliorates STZ-induced learning and memory deficits and neuronal degeneration

**Figure 2:**
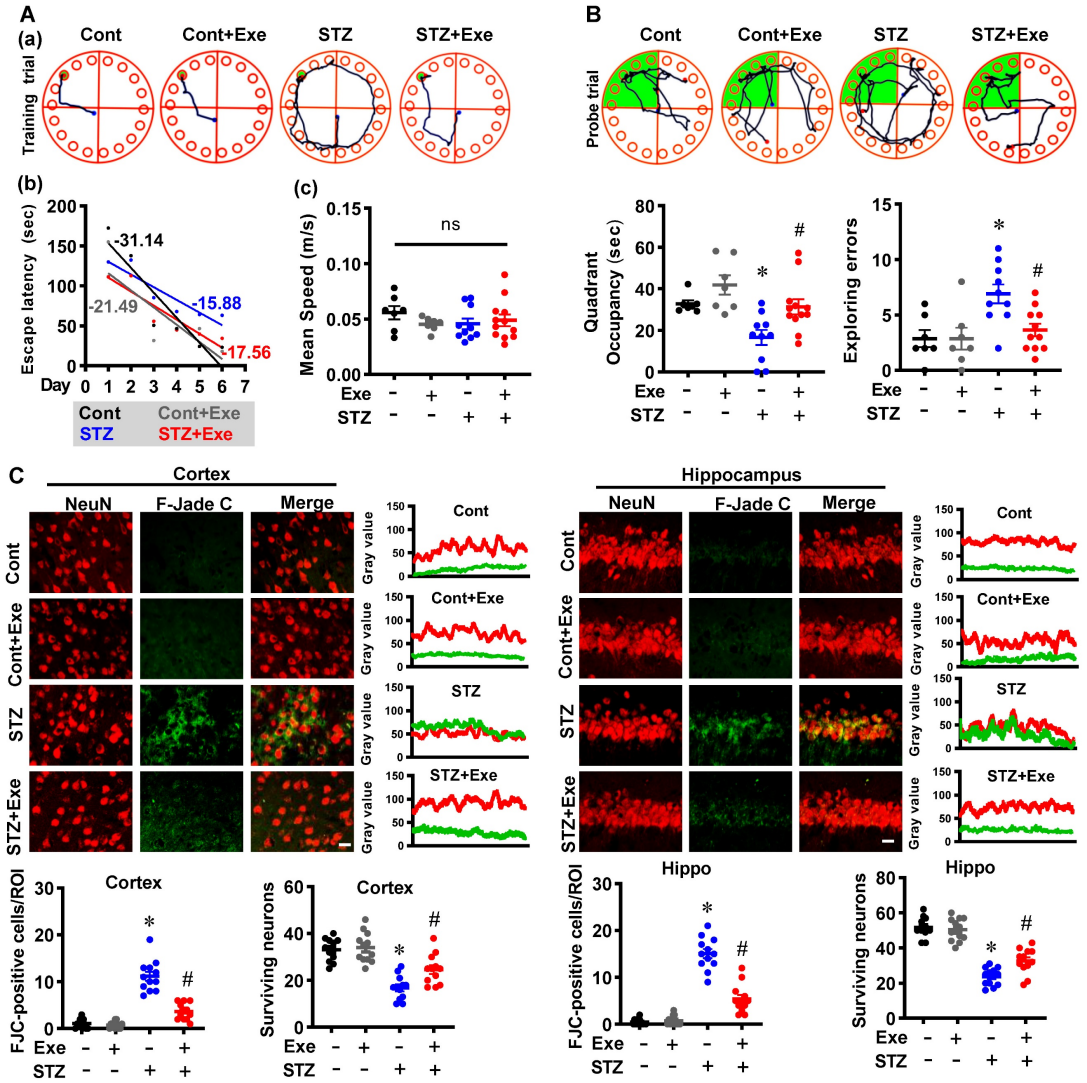
**HIIT ameliorates STZ-induced learning and memory deficits and neuronal degeneration. (A)** Representative tracking plots and results of rats in the training trials of the Barnes maze tests. The slope of line (b) and the mean speed (c) were analyzed. **(B)** Results of the probe test in the Barnes maze tests (n = 7-12). **(C)** Representative immunofluorescence images of NeuN (red) and Fluoro-Jade C staining (green). The scale bar represents 10 μm. Line-scan analysis was performed to analyze the colocalization. The Fluoro-Jade C-positive neurons and surviving neurons were analyzed. Data represent mean ± SEM, n = 12 slices from 6 animals. ^*^P < 0.05 vs. Cont group, ^#^*P* < 0.05 vs. STZ group. ns represents no significant difference.

We first measured the cognitive function and neuronal degeneration in the STZ-induced sporadic Alzheimer's rat model with or without HIIT. The Barnes maze test was performed to assess spatial learning and memory. As shown in **Fig. 2A**, results in the training trials showed that the STZ animals displayed an impaired learning curve compared to control rats. STZ rats with HIIT exhibited an improved learning curve than sedentary STZ rats (**Fig. 2A**). Notably, no significant changes were observed in the escape velocity between groups, indicating that the differences in the learning curve were not due to the speed variations (**Fig. 2A**). In addition, the probe trial results revealed that STZ animals with HIIT spent a longer time than STZ animals without HIIT in the target quadrant where the escape box was located (**Fig. 2B**). Consistent with these results, the STZ rats displayed significantly increased exploring errors during the probe trial than the control and the STZ animals undergoing HIIT (**Fig. 2B**).

Subsequently, F-Jade C staining was employed to label the degenerating neurons. As shown in **Fig. 2C**, the representative images of NeuN and F-Jade C double staining and line-scan analysis showed significantly elevated F-Jade C-positive cells and decreased surviving neurons in the cortex and hippocampus of STZ rats, suggesting enhanced neuronal degeneration following STZ administration. In contrast, STZ rats undergoing HIIT exhibit a significantly alleviated neuronal degeneration and increased numbers of surviving neurons. Similar results were obtained in the transgenic AD mice model, as evidenced by reduced neuronal degeneration following exercise training (**Supplementary Fig. 1A**). These findings indicate that HIIT ameliorates neuronal degeneration in both the STZ-induced sporadic AD rat model and APP/PS1 familial mouse model.

### HIIT reduces neuronal apoptosis and offers robust neuroprotection following STZ administration

**Figure 3:**
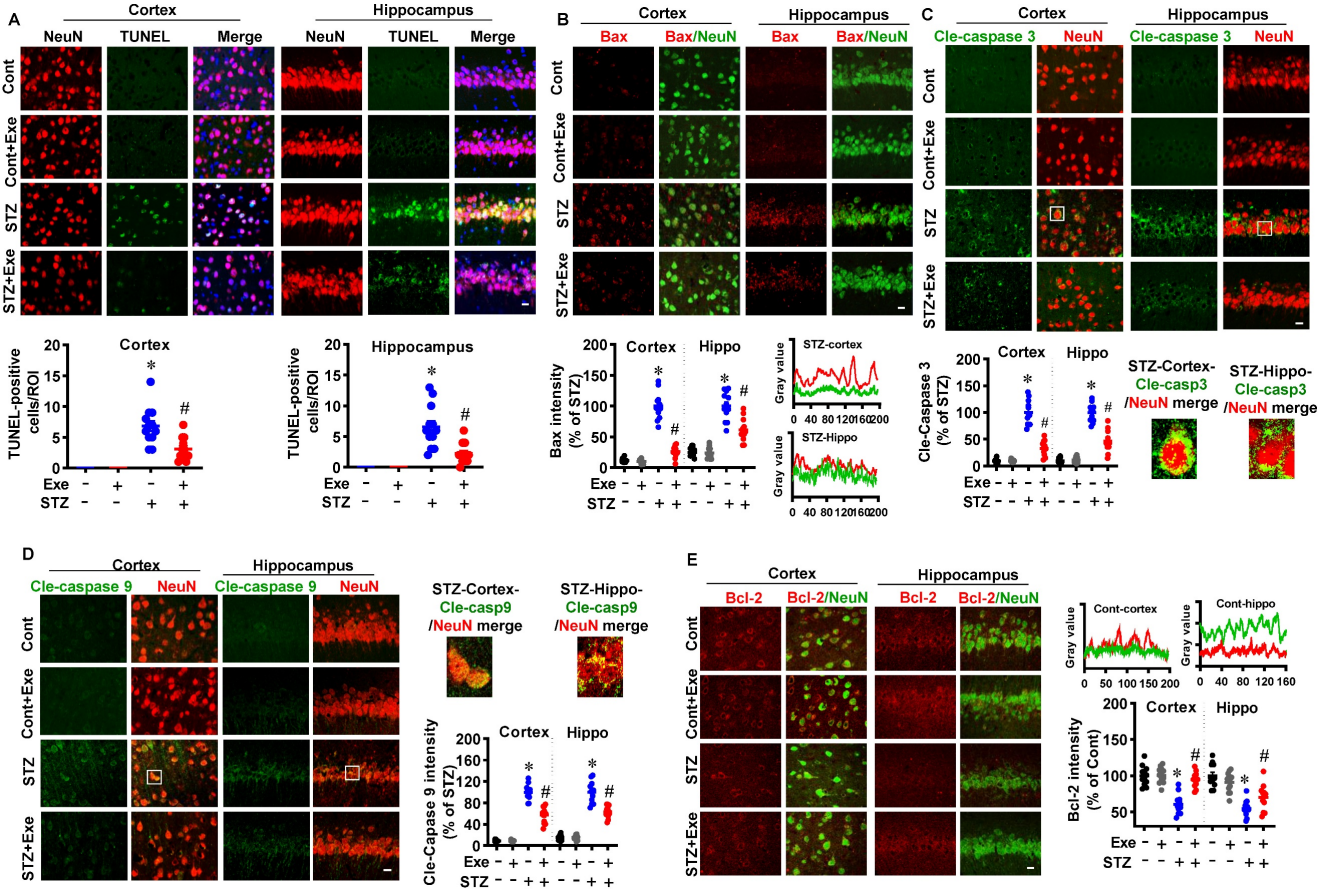
** HIIT reduces neuronal apoptosis following STZ administration. (A)** Representative immunofluorescence images of NeuN (red) and TUNEL staining (green). White arrows indicate TUNEL^+^ neurons. TUNEL-positive cells were counted and analyzed in the cortex and hippocampus.** (B)** Representative confocal images showed NeuN (green) and Bax (red) fluorescent signals in the cortex and hippocampus. Bax intensity was analyzed and shown as a percentage of control. Line-scan analysis of representative images was conducted to analyze the colocalization of Bax and NeuN signals. **(C)** Representative confocal images of Cle-caspase 3 (Green) and NeuN (Red). The white square marked an enlarged view of the area. Cle-Caspase 3 intensity was analyzed as a percentage of STZ. **(D)** Representative confocal images of Cle-caspase 9 (Green) and NeuN (Red). An enlarged view of the area was marked by the white square. Cle-Caspase 9 intensity was analyzed as a percentage of STZ. **(E)** Representative confocal images showed NeuN (green) and Bax (red) fluorescent signals in the cortex and hippocampus. Bax intensity was analyzed and shown as a percentage of control. Line-scan analysis of representative images was conducted to analyze the colocalization of Bax and NeuN signals. The scale bar represents 10 μm. Data represent mean ± SEM, n = 12 slices from 6 animals. ^*^P < 0.05 vs. Cont group, ^#^*P* < 0.05 vs. STZ group.

We next sought to examine the effects of HIIT on neuronal apoptosis and neuroprotection following STZ administration. As shown in **Fig. 3A**, TUNEL staining showed that STZ induced significantly increased TUNEL-positive neurons compared to control animals. In contrast, HIIT alleviated STZ-induced neuronal apoptosis, as evidenced by decreased TUNEL-positive neurons. Similar results were obtained in the transgenic AD mice model. As shown in **Supplementary Fig. 1B**, exercise training ameliorates neuronal apoptosis in transgenic APP/PS1 mice.

To further confirm these results, we also examined the intrinsic apoptosis pathway. As shown in **Fig. 3(B-D)**, the levels of Bax, cleaved caspase-3, and cleaved caspase-9 were significantly increased in the cortex and hippocampus of STZ animals compared with the control and HIIT animals. Consistent with these findings, the level of the anti-apoptotic protein, Bcl-2, was decreased in the STZ animals, whereas HIIT alleviated this decrease (**Fig. 3E**).

**Figure 4:**
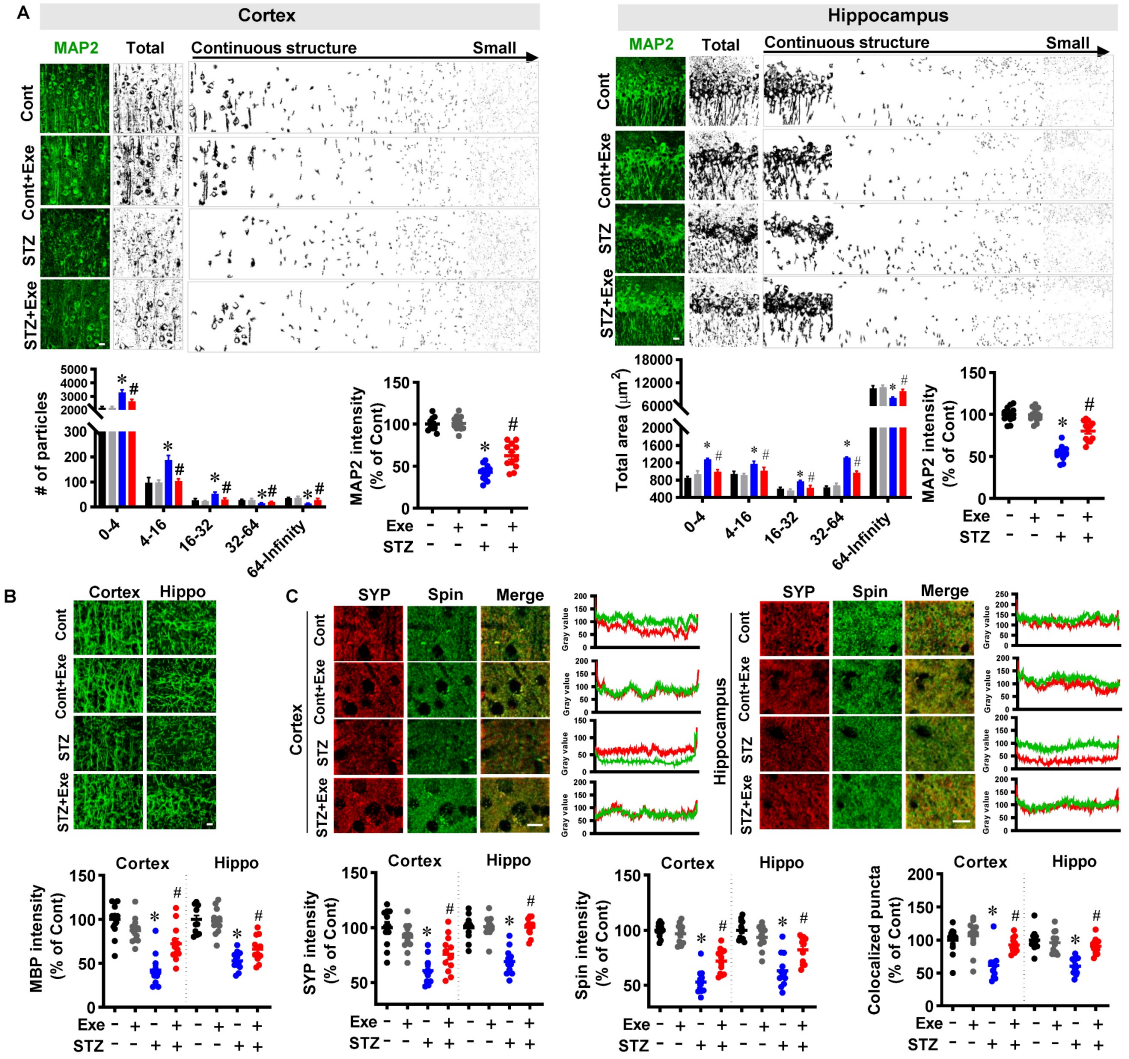
** HIIT alleviates neuronal damage induced by STZ administration. (A)** Representative confocal microscopy images of MAP2. The acquired images of MAP2 were thresholded, median filtered, and binarized using Image J software. The MAP2 segments were size-separated from the continuous structure to small particles. The number of different sizes of particles was analyzed. MAP2 intensity was analyzed as a percentage of control. The scale bar represents 10 μm. The MAP2 segments were area-separated and analyzed. **(B)** Representative confocal microscopy images of MBP. MBP intensity was calculated as a percentage of control. **(C)** Representative immunofluorescence images of synaptophysin (SYP, red, presynaptic marker) and spinophilin (Spin, green, dendritic spine marker). Line-scan analysis was performed to analyze the colocalization of spinophilin and synaptophysin. Immunoactivity intensity analyses of spinophilin and synaptophysin and the colocalized puncta between the two channels were qualified and analyzed using Image J software. The scale bar represents 10 μm. Data represent mean ± SEM, n = 12 slices from 6 animals. ^*^P < 0.05 vs. Cont group, ^#^*P* < 0.05 vs. STZ group.

Moreover, to investigate the effects of HIIT on neuronal injury, MAP2 and MBP staining were performed. As shown in **Fig. 4A**, STZ induced a robust MAP2 fragmentation in the cortex and hippocampus compared to control rats. By contrast, the degree of MAP2 fragmentation was significantly alleviated by HIIT. In line with these findings, STZ rats showed a decreased MAP2 intensity compared to control animals. Intriguingly, the decreased MAP2 intensity in the STZ rats was significantly alleviated by HIIT. Notably, no significant differences were observed between control rats with or without HIIT. Similarly, exercise training preserved the MAP2 and MBP levels in the transgenic APP/PS1 mice (**Supplementary Fig. 2**).

Additionally, as shown in **Fig. 4B**, MBP staining showed that STZ rats exhibited a significantly decreased MBP fluorescent intensity in the cortex and hippocampus compared to the control and HIIT-treated STZ animals. To further confirm the neuroprotective effects of HIIT following STZ administration, we next measured the levels of the dendritic spine marker spinophilin and the presynaptic marker synaptophysin. As shown in **Fig. 4C**, the results showed that the relative fluorescence intensity of synaptophysin and spinophilin was significantly decreased compared to control animals. However, HIIT remarkably preserved the synaptophysin and spinophilin levels in the cortex and hippocampus of STZ rats, suggesting that HIIT alleviated STZ-induced synaptic damage. Consistent with this interpretation, the quantitative analysis of colocalized synaptic puncta suggested that STZ animals undergoing HIIT displayed significantly elevated colocalization between the synaptophysin and spinophilin signals compared with STZ animals, confirming the remarkably alleviated synaptic damage compared with STZ animals.

### HIIT reduces the over-activation of glial cells and induces glial phenotype polarization toward the M2/A2 phenotype following STZ administration

**Figure 5:**
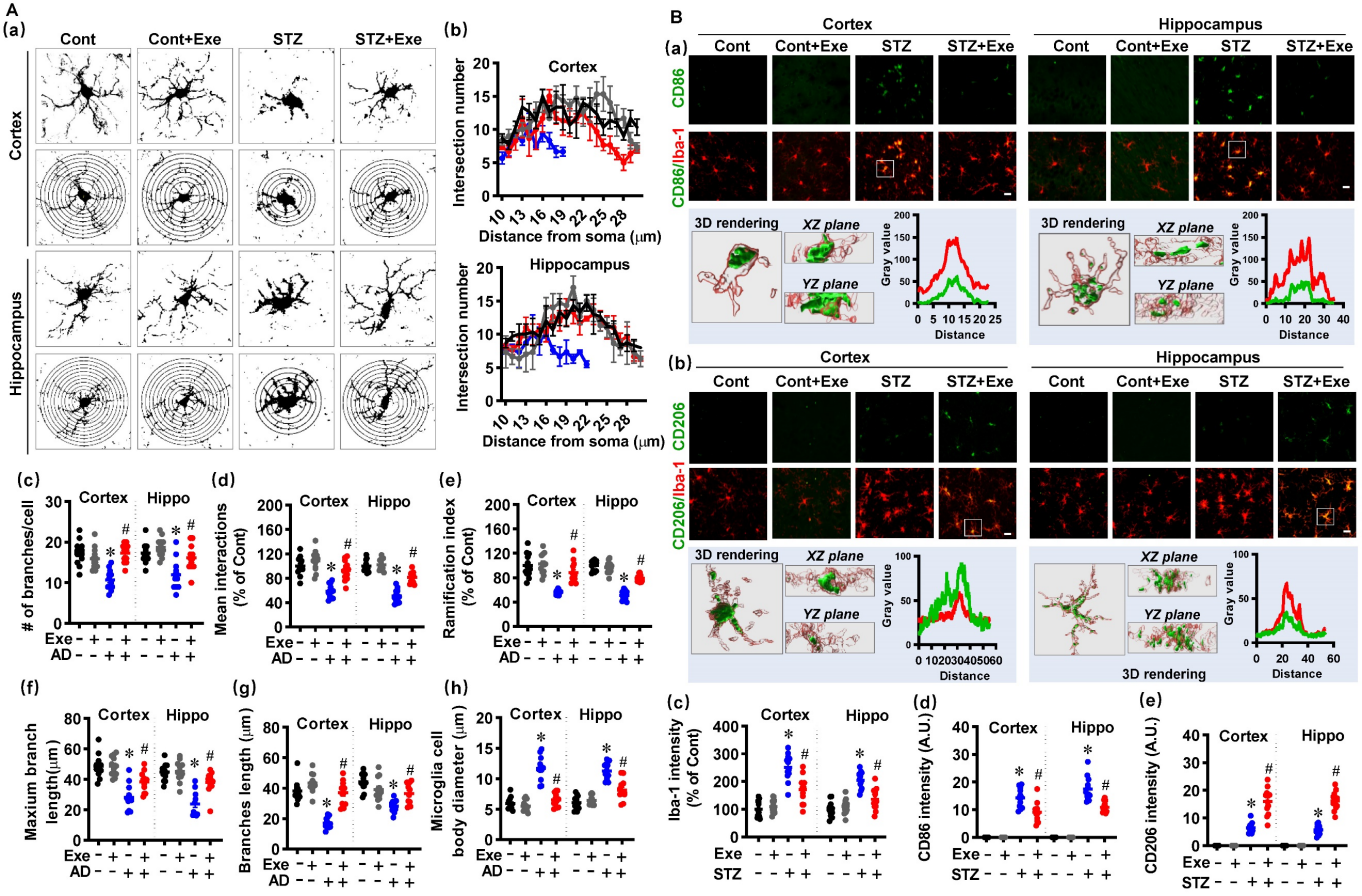
** HIIT reduces the over-activation of microglia and induces microglia polarization toward the M2 phenotype. (A)** Representative images of Iba1^+^ microglia and the Sholl analysis (a). The intersection number per radius over the distance from the cell body was displayed graphically in the curve. (b). The number of branches (c), mean interactions (d), ramification index (e), maximum branch length (f), average branch length (g), and cell body diameter (h) was analyzed. **(B)** Representative confocal microscopy shows co-staining of CD86 (green, M1 marker) and Iba-1 (red). 3D reconstruction and line-scan analysis of the representative cells (white square) in the STZ group were conducted (a). Representative confocal microscopy shows co-staining of CD206 (green) and Iba-1 (red). 3D reconstruction and line-scan analysis of the representative cells (white square) were conducted (b). Iba-1 (c), CD86 (d), and CD206 intensities were analyzed. The scale bar represents 10 μm. Data represent mean ± SEM, n = 12 slices from 6 animals. ^*^P < 0.05 vs. Cont group, ^#^*P* < 0.05 vs. STZ group.

Neuroinflammation contributes to neuronal damage and AD's pathological development 1. Therefore, we investigated the effects of HIIT on the activation and polarization of microglia and astrocyte, the primary innate immune cells in the central nervous system 1. Firstly, the microglial Sholl analysis was performed to assess the complexity and morphology of microglia. As shown in **Fig. 5A (a)**, the microglial branching was measured using concentric circles in Sholl analysis. The STZ rats displayed markedly decreased intersection numbers with the concentric circles. In contrast, HIIT alleviated this decrease in the intersection numbers in the cortex and hippocampus of STZ animals (**Fig. 5A(b)**). Consistent with these findings, STZ rats also exhibited significant reductions in the number of branches (**Fig. 5A(c)**), mean interactions (**Fig. 5A(d)**), ramification index (**Fig. 5A(e)**), maximum branch length (**Fig. 5A(f)**), and branch length (**Fig. 5A(g)**), and the increased size of the microglia cell body (**Fig. 5A(h)**) in the cortex and hippocampus of STZ rats. Intriguingly, HIIT suppressed these changes, suggesting that HIIT alleviated the over-activation of microglia in the cortex and hippocampus of STZ rats.

In brain injury and neurodegenerative diseases, microglia can be polarized into M1 pro-inflammatory and M2 anti-inflammatory phenotypes 1, 26. As shown in **Fig. 5B(a)**, double staining of CD86, a marker of M1 phenotype, with Iba-1 showed markedly increased CD86-positive microglia in the cortex and hippocampus after STZ administration. In contrast, HIIT-treated animals displayed significantly decreased CD86-positive microglia. 3D rendering and line-scan analysis confirmed the colocalization of CD86 and Iba-1. Moreover, we measured the double staining of CD206, an M2 phenotypic marker, with Iba-1 and found a remarkable increase in CD206-positive microglia (**Fig. 5B(b)**), suggesting that HIIT enhanced the microglia polarization from M1 toward M2 phenotype. Quantitative analysis of the fluorescent intensity of Iba-1, CD86, and CD206 confirmed these findings (**Fig. 5B(c-e)**).

**Figure 6:**
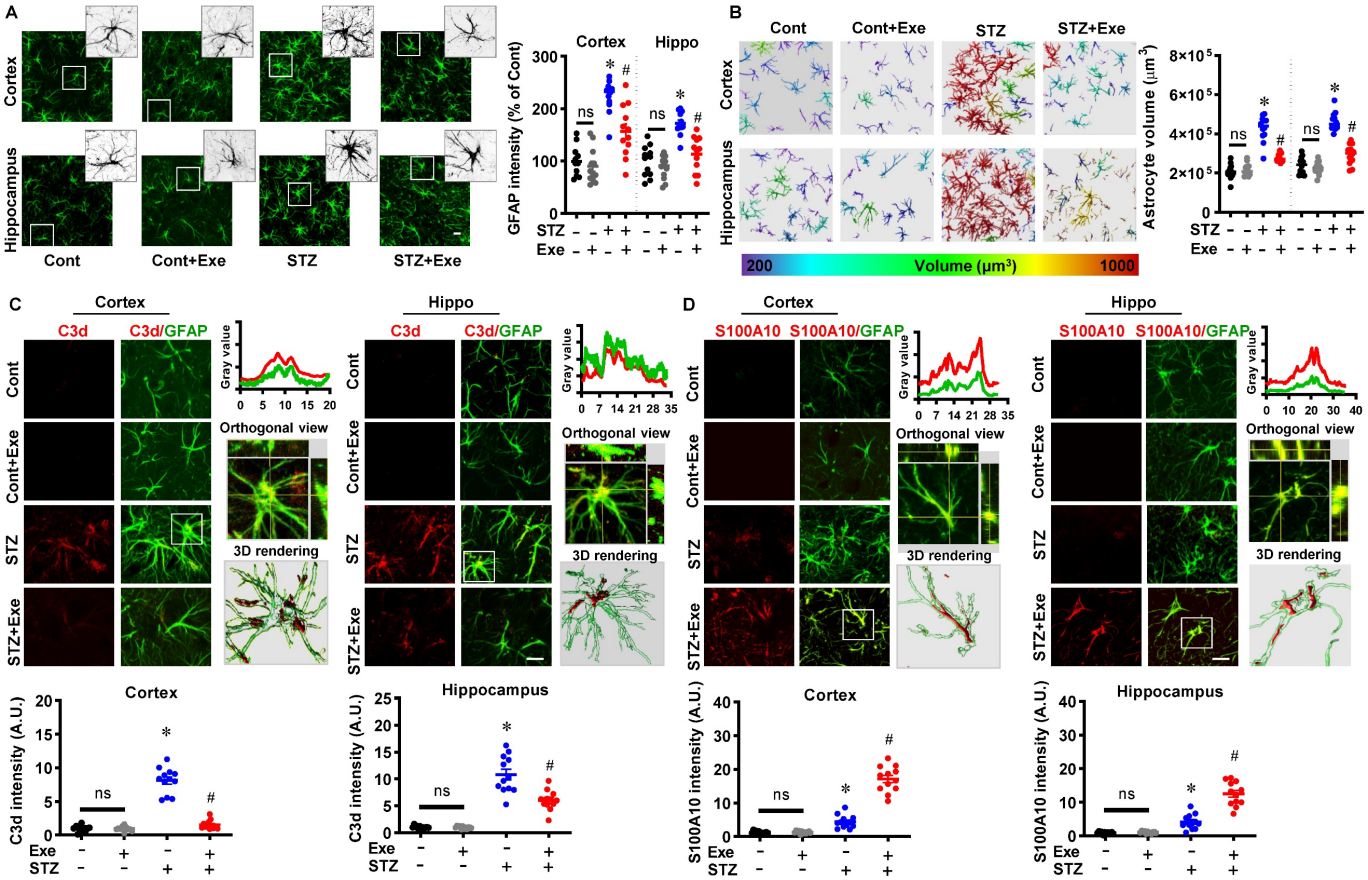
** HIIT reduces the over-activation of astrocytes and induces astrocyte polarization toward the A2 phenotype. (A)** Representative images of GFAP^+^ astrocyte. An enlarged view of the white square-marked astrocyte was shown at the top right corner. The GFAP intensity was quantified and analyzed. **(B)** Representative 3D reconstruction images of astrocytes with the cellular volumes distinguished with different colors. Astrocyte volumes were quantified and analyzed. **(C)** Representative confocal microscopy shows co-staining of C3d (red, A1 marker) and GFAP (green). Line-scan analysis, orthogonal view, and 3D reconstruction of the representative cells (white square) in the STZ group were conducted to confirm the colocalization of C3d and GFAP. C3d intensities in the cortex and hippocampus were measured and analyzed. **(D)** Representative confocal microscopy shows co-staining of S100A10 (red, A2 marker) and GFAP (green). Line-scan analysis, orthogonal view, and 3D reconstruction of the representative cells (white square) in the exercise group were conducted to confirm the colocalization of S100A10 and GFAP. S100A10 intensities in the cortex and hippocampus were measured. The scale bar represents 10 μm. Data represent mean ± SEM, n = 12 slices from 6 animals. ^*^P < 0.05 vs. Cont group, ^#^*P* < 0.05 vs. STZ group.

Similarly, astrocyte activation and phenotype polarization have been implicated in AD progression 4. We next investigated the changes in astrocyte activation and phenotype polarization. As shown in **Fig. 6A**, STZ administration induced significantly increased GFAP intensity in the cortex and hippocampus compared to control animals, which was alleviated by HIIT treatment. The increased astrocyte volume in the STZ group was also attenuated by HIIT, which further confirmed that HIIT impaired the over-activation of astrocytes following STZ administration (**Fig. 6B**). Similar to microglia, astrocyte polarization from the pro-inflammatory A1 phenotype to the neuroprotective A2 phenotype was detected in the HIIT-treated animals, as evidenced by significantly increased S100A10 levels (an A2 phenotype marker) and a significant reduction in C3d levels (an A1 phenotype marker) (**Fig. 6 (C&D)**). Similar results were obtained in the transgenic AD mouse model (**Supplementary Fig. 3**).

### HIIT preserves astrocyte p-AQP4 and n-AQP4 polarity

**Figure 7:**
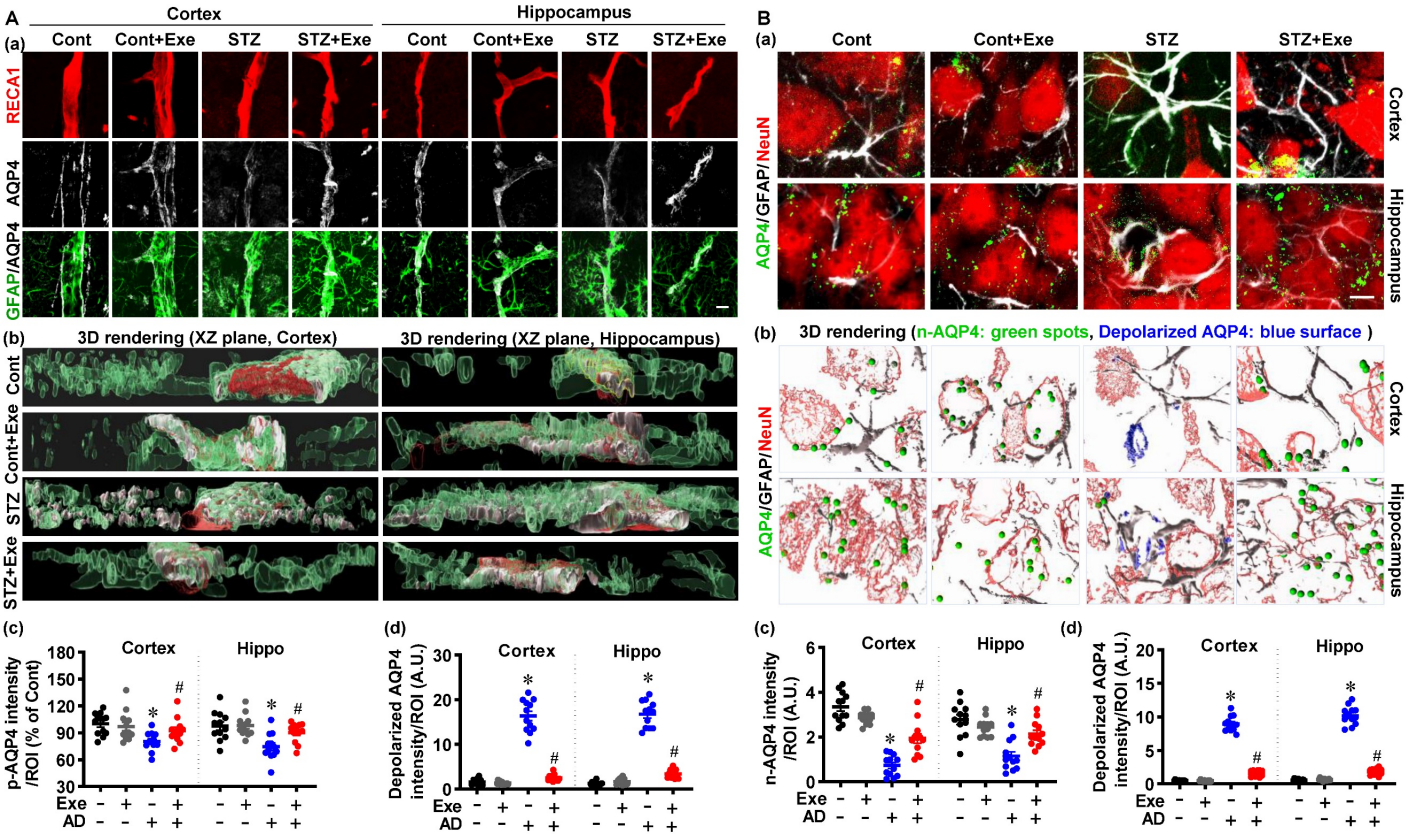
** HIIT preserves astrocyte p-AQP4 and n-AQP4 polarity. (A)** Confocal immunofluorescence triple staining for RECA1 (red), AQP4 (white), and GFAP (green) (a). XZ-plane view of the 3D rendering images (b). p-AQP4 and depolarized AQP4 intensities were analyzed (c and d). The scale bar represents 10 μm. **(B)** Confocal immunofluorescence triple staining for NeuN (red), AQP4 (green), and GFAP (white) (a). 3D rendering images of the NueN/GFAP/AQP4. The green ball represents n-AQP4, and the blue surface represents depolarized AQP4 (b). n-AQP4 and depolarized AQP4 intensities were analyzed (c and d). A.U. indicates arbitrary units. The scale bar represents 5 μm. Data represent mean ± SEM, n = 12 slices from 6 animals. ^*^P < 0.05 vs. Cont group, ^#^*P* < 0.05 vs. STZ group.

As mentioned previously, astrocyte p-AQP4 and n-AQP4 polarity are indispensable for extracellular Aβ and abnormal intracellular tau removal 6. Therefore, we next examined the effects of HIIT upon astrocyte p-AQP4 and n-AQP4 in the cortex and hippocampus of STZ-injected rats. Representative co-staining images of GFAP, AQP4, and RECA1 (an endothelial cell surface marker) showed significantly decreased levels of p-AQP4 and increased depolarized AQP4 in the cortex and hippocampus of STZ rats (**Fig. 7A(a)**). The 3D-rendered images confirmed the distribution of p-AQP4 and depolarized AQP4 in **Fig. 7A (b)**. Next, the n-AQP4 and depolarized AQP4 were assessed by co-staining of AQP4 with NeuN and GFAP. As shown in **Fig. 7B (a)**, the polarized n-AQP4 was significantly decreased in the cortex and hippocampus of STZ-treated animals compared to control animals. In contrast, HIIT ameliorated this decrease in the cortex and hippocampus of STZ animals. Consistent with the changes of p-AQP4 in **Fig. 7A**, the depolarized AQP4 around neurons was significantly increased in STZ animals and attenuated by HIIT (**Fig. 7B**). The 3D-rendered images confirmed the distribution of p-AQP4 and depolarized AQP4 (**Fig. 7B(b)**). No significant differences were detected between control rats with or without HIIT. Findings presented in **Supplementary Fig. 4** confirm that exercise training preserves astrocyte p-AQP4 and n-AQP4 polarity in the cortex of the transgenic APP/PS1 mice.

### HIIT-induced AQP4 polarized distribution correlates closely with astrocyte phenotype without affecting AQP4 expression

**Figure 8:**
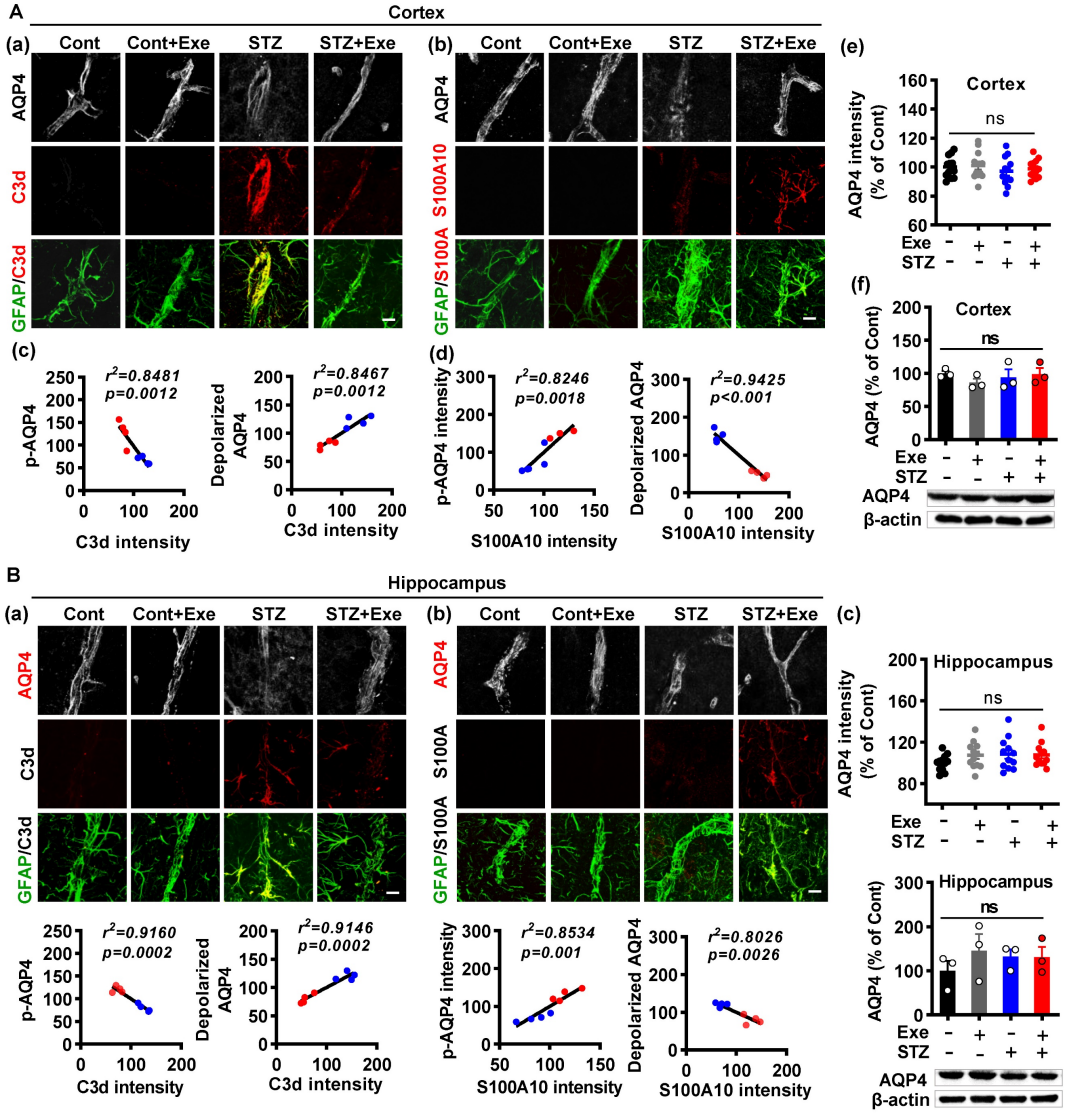
** HIIT-induced AQP4 polarized distribution correlates closely with astrocyte phenotype without affecting AQP4 expression. (A)** Representative immunofluorescence images of AQP4 (white), C3d (red), and GFAP (green) in the cortex (a). Representative immunofluorescence images of AQP4 (white), S100A10 (red), and GFAP (green) in the cortex (b). Linear regression analysis of the association between AQP4 (p-AQP4 or depolarized AQP4) and C3d (c) or S100A10 (d). The levels of AQP4 in the cortex were measured by quantitative analysis of AQP4 immunofluorescence intensity (e, n = 12 slices from 6 animals) and western blot (f, n = 3). Data represent mean ± SEM. The scale bar represents 10 μm. **(B)** Representative immunofluorescence images of AQP4 (white), C3d (red), and GFAP (green) in the hippocampus (a). Representative immunofluorescence images of AQP4 (white), S100A10 (red), and GFAP (green) in the hippocampus (b). Linear regression analysis of the association between AQP4 (p-AQP4 or depolarized AQP4) and C3d (c) or S100A10 (d). The levels of AQP4 in the hippocampus were measured by quantitative analysis of AQP4 immunofluorescence intensity (e, n = 12 slices from 6 animals) and western blot (f, n = 3). Data represent mean ± SEM. The scale bar represents 10 μm. ^*^P < 0.05 vs. Cont group, ^#^*P* < 0.05 vs. STZ group.

As both the astrocyte polarization and AQP4 polarity were affected by HIIT, we examined the relationship between the AQP4 polarity and astrocyte phenotype polarization. As shown in **Fig. 8A (a),** triple immunofluorescence labeling of GFAP, C3d, and AQP4 displayed that the A1 astrocytes had remarkably elevated depolarized AQP4 in the cortex of STZ animals, which was dramatically ameliorated in HIIT-treated STZ rats. In line with this finding, A2 astrocytes also showed decreased depolarized AQP4 in the STZ animals undergoing HIIT treatment (**Fig. 8A(b)**). To determine the relationship between AQP4 polarity and astrocyte phenotype, we performed a linear regression analysis to examine p-AQP4 or depolarized AQP4 with C3d and S100A10. As shown in **Fig. 8A (c)**, statistical analysis revealed a negative regression between p-AQP4 and C3d intensity and a positive regression between depolarized AQP4 and C3d intensity. In contrast, linear regression showed a significant positive correlation between p-AQP4 and S100A10 and a negative regression between depolarized AQP4 and S100A10 (**Fig. 8A(d)**). Notably, HIIT and STZ did not cause any change in AQP4 levels of the cortex and hippocampus (**Fig. 8A(e and f)**). Analysis of the hippocampus obtained similar results shown in **Fig. 8B**.

### HIIT attenuates amyloid load and abnormal tau hyperphosphorylation through polarized AQP4 and kidney-mediated clearance

**Figure 9:**
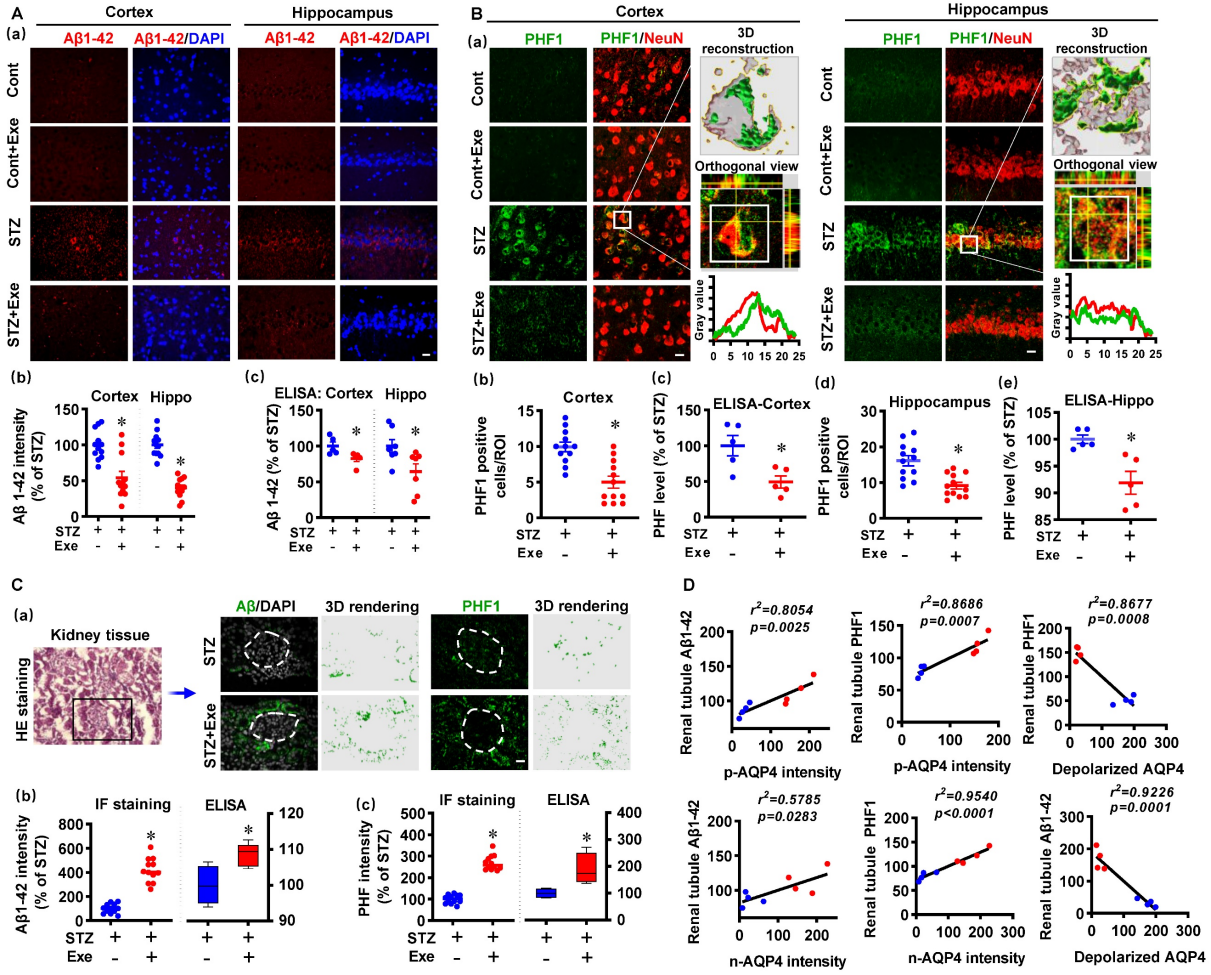
** HIIT attenuates amyloid load and abnormal tau hyperphosphorylation through kidney-mediated clearance and polarized AQP4. (A)** Representative immunofluorescence images of Aβ1-42 (red) and DAPI (blue) in the cortex and hippocampus (a). The Aβ1-42 intensities in the cortex and hippocampus were quantified and analyzed (b, n = 12 slices from 6 animals). The scale bar represents 10 μm. The levels of Aβ1-42 in the cortex and hippocampus were measured by ELISA (c, n = 5). The Aβ1-42 intensity was presented as a percentage of the STZ group. **(B)** Representative immunofluorescence images of PHF1 (green) and NeuN (red) in the cortex and hippocampus (a). 3D reconstruction of the neuron (white square), orthogonal view, and line-scan analysis in the STZ group were conducted to confirm the colocalization of PHF1 and NeuN (a). Numbers of PHF1 positive cells in the region of interest (b and d, n =12 slices from 6 animals). The PHF level in the cortex and hippocampus were measured by ELISA (c and e, n = 5). **(C)** Representative immunofluorescence and 3D rendering images of Aβ1-42 and PHF1 in the kidney tissue (a). The images of HE staining showed the glomerulus and kidney tubule marked by a box where the immunofluorescence images were taken (a). Immunofluorescence intensity and ELISA analysis results of Aβ1-42 (b) and PHF (c) were displayed as a percentage of the STZ group.** (D)** Linear regression analysis of the association between AQP4 (n-AQP4, p-AQP4, and depolarized AQP4) and the levels of Aβ or PHF1 in the renal tubule. *P* < 0.05 vs. STZ group.

We then analyzed the levels of Aβ1-42 and hyperphosphorylated tau in the cortex and hippocampus. As shown in **Fig. 9A (a&b)**, the cortex and hippocampus of STZ animals exhibited dramatically increased Aβ1-42, which was attenuated by HIIT. A specific Aβ (1-42) ELISA test confirmed this result (**Fig. 9A(c)**). In addition, the findings presented in **Supplementary Fig. 5** confirm that exercise training attenuates amyloid load in the cortex and hippocampus in the transgenic APP/PS1 mice. Similarly, increased PHF was detected in the cortex and hippocampus of STZ animals (**Fig. 9B(a)**). The colocation of PHF and NeuN in the STZ animals was confirmed by 3D reconstruction and line-scan analysis (**Fig. 9B (a)**). HIIT significantly ameliorated PHF in the cortex and hippocampus following STZ administration (**Fig. 9B (b and d)**). These findings in PHF were confirmed by PHF ELISA tests (**Fig. 9B (c and e)**).

Because kidneys are the primary excretory organs involved in removing internal metabolic wastes and AQP4-mediated glymphatic drainage 6, we finally examined Aβ1-42 and PHF in the kidney tissue using immunofluorescence staining and ELISA tests. As shown in **Fig. 9C**, Aβ1-42 and PHF1 were detected in the renal tubule of STZ animals with and without HIIT. Intriguingly, HIIT enhanced the drainage of Aβ1-42 and abnormal tau, as evidenced by significantly increased Aβ and PHF1 in the renal tubule. Additionally, the linear regression analysis showed that the levels of Aβ1-42 and PHF1 in the renal tubule were positively correlated with p-AQP4 and n-AQP4 intensity, suggesting the polarized AQP4 contributed to the drainage of Aβ1-42 and PHF (**Fig. 9D**). In contrast, linear regression showed a significant negative correlation between the depolarized AQP4 and Aβ1-42 and PHF1 in the renal tubule, respectively (**Fig. 9D**).

In addition, to confirm the effects of HIIT on glymphatic transport and analyze whether Aβ detected in the kidney was from the brain, a CSF tracer diffusion experiment with the fluorescently labeled Aβ (TAMRA-Aβ) was performed. As shown in **Supplementary Fig. 6,** the area covered by the tracer was significantly increased in the STZ animal with exercise training, suggesting HIIT promotes glymphatic transport. Additionally, as shown in **Supplementary Fig. 7**, the fluorescently labeled Aβ was significantly increased in the kidney of STZ-induced AD animals with HIIT training, the Aβ i.c.v injected into the brain could be removed from the brain tissue through the glymphatic system and the kidney.

## Discussion

Our study demonstrates that HIIT alleviates cognitive dysfunction in STZ-induced AD-like rat models and provides neuroprotection against neurodegeneration, neuronal damage, and neuronal loss. Notably, HIIT improved the drainage of abnormal tau and Aβ from the cortex and hippocampus via the glymphatic system to the kidney. Further mechanistic studies revealed that the beneficial effects of HIIT on AD might be due, in part, to the polarization of glial cells from a neurotoxic phenotype towards a neuroprotective phenotype. Furthermore, an intriguing finding of our study is that the polarized distribution of AQP4 was strongly correlated with astrocyte phenotype. We found A2 phenotype exhibited more evident AQP4 polarization than the A1 phenotype. This finding suggests that the beneficial effects of HIIT on the clearance of Aβ and abnormal tau, at least in part, is due to the regulation of the astrocyte phenotype-associated AQP4 polarization. Our studies provided additional evidence about HIIT's potential use in promoting Aβ and p-tau clearance and clarified the contributing mechanisms.

The effects of various types of physical exercise have been investigated in AD, including aerobic exercise and resistance exercise 22, 29, 30. HIIT, a time-saving and highly efficient exercise modality with a short burst of high-intensity exercise training, has recently attracted increasing interest 21. Previous studies indicated that HIIT exerts neuroprotective effects and alleviates memory deficits in AD 31, 32. Using an STZ-induced sporadic Alzheimer's rat model, we detected spatial learning and memory deficits in STZ-administrated rats 22. The STZ-induced AD model is a widely used and well-established model of sporadic AD 33-35. It presents excessive production Aβ, p-tau, neuronal degeneration, neuroinflammation, and oxidative stress 33-35. However, the STZ-induced AD rat model does not exhibit amyloid plaques. Therefore, we also confirmed our key findings using the transgenic AD mouse model (APP/PS1), a widely used familial AD mouse model with Aβ plaques deposit. In line with previous studies, impaired spatial learning and memory were significantly improved by HIIT. Moreover, we measured neuronal degeneration, apoptosis, and damage with or without HIIT. Consistent with earlier research, STZ rats exhibit substantial neuronal damage, apoptosis, and neurodegeneration following STZ administration 22. In contrast, HIIT significantly alleviates these changes suggesting that, as an emerging trend in public health promotion, HIIT confers a similar neuroprotective effect against cognitive dysfunction in an AD-like model as aerobic exercise 22, 30.

Neuroinflammation has been inextricably associated with the initiation and progression of AD 1, 14, 22. In AD, neuroinflammation is characterized by the hyperactivation of glial cells, which triggers an excessive release of inflammatory cytokines and exacerbates Aβ accumulation and tau hyperphosphorylation 1, 36. For example, inflammatory cytokines, including IL-1β, TNF-α, IL-6, and IL-12, are elevated from the early stage of AD and contribute to the progression of pathology 36. Animal studies and clinical trials demonstrated that anti-inflammatory approaches could alleviate AD progression, although the beneficial effects are limited and sometimes show side effects 37, 38. Glial cells, including microglia and astrocyte, plays a major role in the inflammatory response of AD 1. In response to the Aβ accumulation and tau pathology at the early stages of AD, activated microglia are involved in Aβ phagocytosis. However, sustained microglia activation may cause the loss of phagocytotic function and neuroinflammation 39. In agreement, we observed the overactivated microglia in the cortex and hippocampus of STZ animals, which was strongly ameliorated by HIIT, suggesting HIIT could alleviate microglia-mediated excessive neuroinflammation in AD. Astrocytes also exhibited similar changes following STZ administration and HIIT treatment. Additionally, previous studies found that the activated microglia and astrocyte in AD could be polarized to neurotoxic phenotype M1 and A1, respectively 40. Consistent with previous studies, we detected M1 and A1 polarization in the cortex and hippocampus of STZ-treated animals. Intriguingly, HIIT promotes the polarization of glial cells from the neurotoxic phenotype (M1 and A1) toward the neuroprotective phenotype (M2 and A2). These findings suggest that HIIT could alleviate neuroinflammation and regulate the phenotype of glial cells in STZ-induced AD animal models.

As mentioned previously, the efficiency of the glymphatic system depends on AQP4 8, 9. The changes of AQP4 are extensively studied in various brain injuries 41. The injury severity and the duration after the injury affect AQP4 expression in different brain injury models 41. For instance, AQP4 expression was elevated in a transient middle cerebral artery occlusion (tMCAO) mouse model, and AQP4 knockout alleviates tMCAO-induced brain injury 41, 42. In contrast, the AQP4 expression was decreased at 24 h following permanent MCAO (pMCAO), and AQP4 knockout exacerbates brain injury 41, 43. Accumulating evidence suggests that AQP4 is a potential drug target in neurological disorders 44. Several pharmacologic agents targeting AQP4 have been developed to alleviate brain injury 41. For example, Acetazolamide and TGN-020, nonselective agents, were designed to inhibit AQP4 41, 45. AER-270 and AER-271 have been identified to selectively inhibit AQP4 and improve neurological outcomes in the tMCAO animal model 41, 46. Although several promising results have been obtained in previous studies, the neuroprotective effects of these pharmacologic agents are limited in specific animal models 41. These findings suggest although AQP4 is a promising target for neurological disorders, more studies are still needed.

In AD, AQP4 expression and distribution in astrocytes are essential for efficiently clearing neuronal wastes such as Aβ and abnormal tau 6. Under physiological conditions, astrocyte AQP4 is distributed on endfoot membranes facing vascular (p-AQP4) or neurons (n-AQP4). However, the AQP4 in AD exhibited a depolarized distribution under pathological conditions with increased distribution on parenchymal processes 47. Emerging evidence suggests astrocyte AQP4 polarization is implicated in AD pathology 6. AQP4 depolarization impairs the efficiency of the glymphatic system in Aβ and tau clearance 6. A previous study found that five weeks of voluntary wheel running promoted the influx of CSF with fluorescent tracers in the cortex and the middle cerebral territory in awake-behaving young mice, suggesting exercise training enhances glymphatic influx 48. Aged mice performed 6-week voluntary wheel training to attenuate Aβ accumulation, neuroinflammation, and synaptic dysfunction, possibly due to the increased astrocytic AQP4 expression and AQP4 polarization 49. A recent study found that APP/PS1 mice with 2 months of voluntary wheel exercise presented a significantly decreased Aβ accumulation and perivascular AQP4 mislocalization. However, AQP4 knockout APP/PS1 mice exhibited glymphatic dysfunction and increased Aβ load in the brain, suggesting the essential of the AQP4-dependent glymphatic system in mediating exercise's beneficial role for AD 50. In the current study, we demonstrated that STZ administration induced depolarized distribution of p-AQP4 and n-AQP4 in the cortex and hippocampus. Interestingly, the polarized distribution of AQP4 exhibited a significant positive correlation with the expression of A2 phenotype markers and displayed a significant negative correlation with the marker of the A1 phenotype. The phenotype polarization from A1 to A2 regulated by HIIT is accompanied by preserved polarized AQP4, suggesting that A1 astrocytes may exhibit a depolarized distribution of AQP4 and AQP4 on A2 astrocytes tend to display a polarized distribution. However, more studies regarding its underlying mechanisms are still needed. In addition to the AQP4 distribution, the AQP4 expression is indispensable for the normal function of the glymphatic system. Previous studies demonstrated that AQP4 deficiency aggravates Aβ accumulation and impairs abnormal tau clearance 6, 51. It is worth noting that STZ administration and HIIT did not affect the expression of AQP4. These findings suggest that HIIT only involves the distribution of AQP4 rather than its levels. Compared with previous studies, our study analyzed the effects of HIIT on p-AQP4 and n-AQP4 in removing Aβ and abnormal tau. We also provide evidence for the first time that astrocyte phenotypes are closely associated with AQP4 polarization.

As mentioned previously, one of the most crucial functions of the glymphatic system is draining Aβ and p-tau 6. Depolarized distribution of AQP4 aggravates Aβ accumulation and impairs abnormal tau clearance 52. In agreement with previous studies and the changes in AQP4 distribution 52, we detected significantly increased Aβ and p-tau levels in the renal tubules following HIIT treatment. Intriguingly, the Aβ and p-tau were not seen in the glomerulus because the rats were perfused with saline before sample collection. Our findings are consistent with a previous study, wherein they demonstrated that physiological clearance of Aβ by the kidney was involved in the development of AD pathogenesis 53. Furthermore, interestingly, the levels of Aβ1-42 and p-tau in the renal tubule were positively correlated with the polarized AQP4 levels but negatively correlated with depolarized AQP4, suggesting the physiological clearance of Aβ and p-tau by the kidney is closely associated with AQP4-mediated clearance in the glymphatic system. Finally, it is worth noting that only male animals were used in our study because the female animal is more complex due to the impact of sex and sex hormones 54, 55. However, the effects of sex differences on the glymphatic system and astrocyte phenotype-associated AQP4 polarization deserve further investigation, which is one of the limitations of our study.

## Conclusions

**Figure 10:**
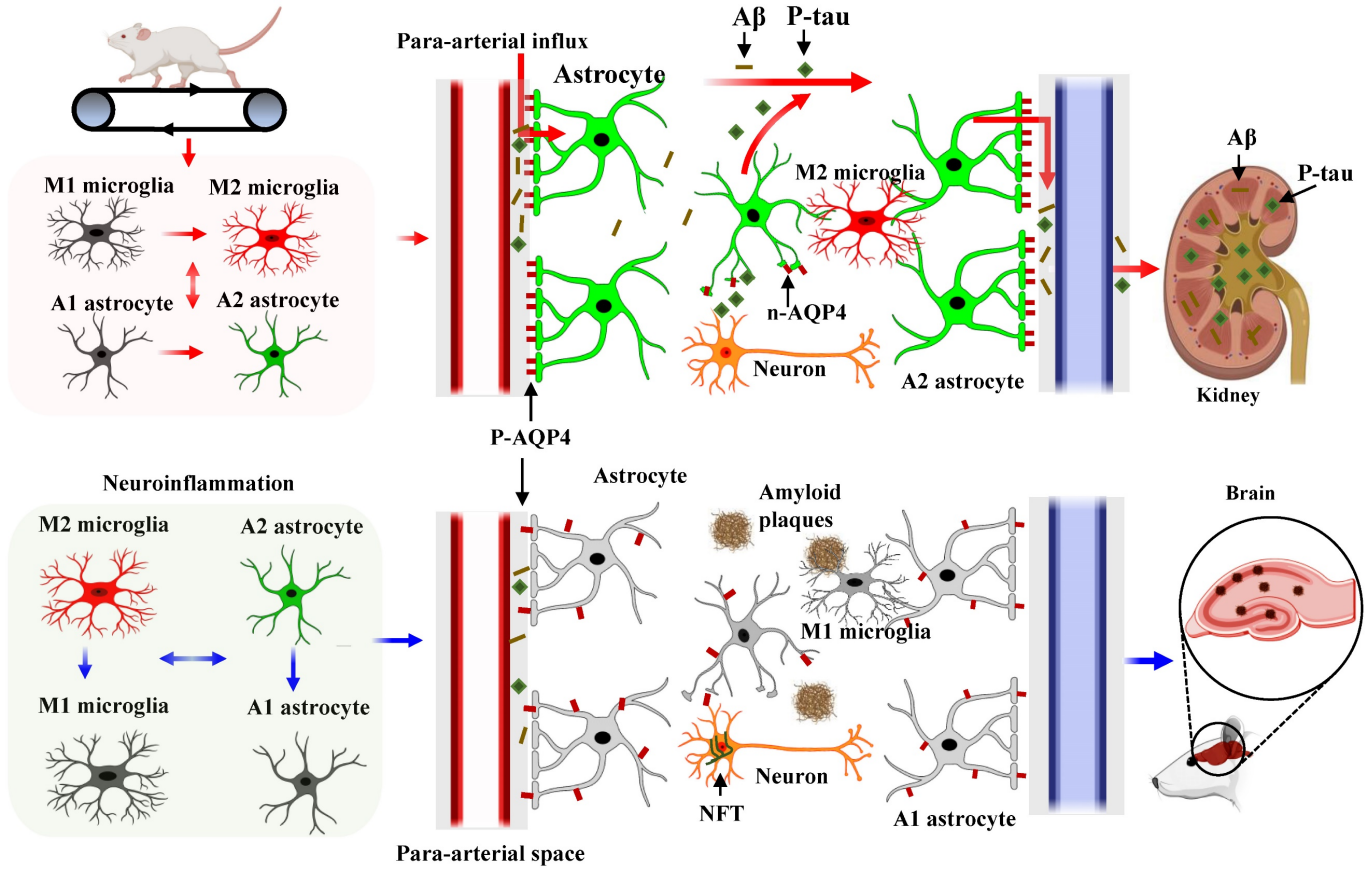
** Diagram of the mechanism of action of HIIT on Aβ and p-tau clearance in STZ-induced AD-like rat models.** HIIT promotes astrocyte polarization from A1 to the A2 phenotype, wherein the AQP4 exhibits polarized distribution in the A2 phenotype and depolarizes in the A1 phenotype. The polarized AQP4 promotes Aβ and p-tau clearance from the brain tissue through the glymphatic system and the kidney.

In conclusion, our study demonstrates that HIIT alleviates AD pathology, including improving learning and memory and reducing neuronal damage, loss, and neurodegeneration. We conclude that the glial cell phenotype polarization and the polarized distribution of astrocyte AQP4 contribute to the beneficial effects of HIIT in AD. Additionally, we provide the first evidence that astrocyte AQP4 distribution is closely related to astrocyte phenotype polarization. As shown in **Fig. 10**, HIIT promotes astrocyte polarization from A1 to the A2 phenotype, wherein the AQP4 exhibits polarized distribution in the A2 phenotype and depolarizes in the A1 phenotype. The polarized AQP4 promotes Aβ and p-tau clearance from the brain tissue through the glymphatic system and the kidney. Overall, these findings significantly advance our understanding of the beneficial role of HIIT in AD and the polarized distribution of AQP4 in different phenotypes of astrocytes.

## Supplementary Material

Supplementary figures.

## Abbreviations

AD: Alzheimer's disease
STZ: streptozotocin (STZ)
ELISA: enzyme-linked immunosorbent assay
Aβ: β-amyloid
NFT: neurofibrillary tangles
CSF: cerebrospinal fluid
ISF: interstitial fluid
AQP4: aquaporin channel 4
HIIT: High-intensity interval training
IACUC: Institutional Animal Care and Use Committee
i.c.v.: intracerebroventricular
PVDF: polyvinylidene difluoride
SDS: sodium dodecyl sulfate-polyacrylamide
BSA: bovine serum albumin
TMB: 3,3′,5,5-tetramethylbenzidine
tMCAO: transient middle cerebral artery occlusion
pMCAO: permanent middle cerebral artery occlusion
