# Supplementary material for: High-intensity interval training ameliorates Alzheimer's disease-like pathology by regulating astrocyte phenotype-associated AQP4 polarization
Source: Theranostics. 2023 Jun 4;13(10):3434–50. doi: 10.7150/thno.81951 (PMC10283053; doi:10.7150/thno.81951)
Supplement: Supplementary file 1 — Supplementary figures. [file thnov13p3434s1.pdf]

Supplementary Figures

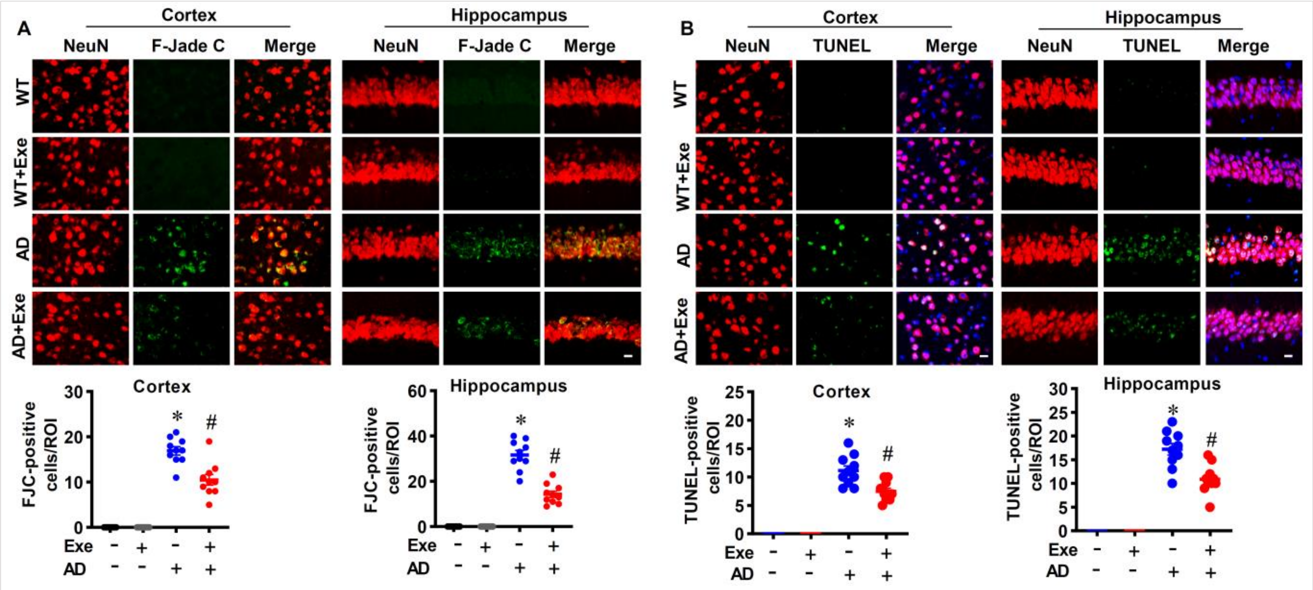

Supplementary Fig. 1. Exercise training ameliorates neuronal degeneration and apoptosis in transgenic APP/PS1 mice. (A) Representative immunofluorescence images of NeuN (red) and Fluoro-Jade C staining (green). The scale bar represents 10 μm. The Fluoro-Jade C-positive neurons were analyzed. Data represent mean ± SEM, n = 10 slices from 5 animals. $^{*}P < 0.05$ vs. Cont group, $^{\#}P < 0.05$ vs. STZ group. ns represents no significant difference. **(B)** Representative immunofluorescence images of NeuN (red) and TUNEL staining (green). TUNEL-positive cells were counted and analyzed in the cortex and hippocampus. ROI indicates the region of interest. The scale bar represents 10 μm. Data represent mean ± SEM, n = 10 slices from 5 animals. $^{*}P < 0.05$ vs. WT group, $^{\#}P < 0.05$ vs. AD group.

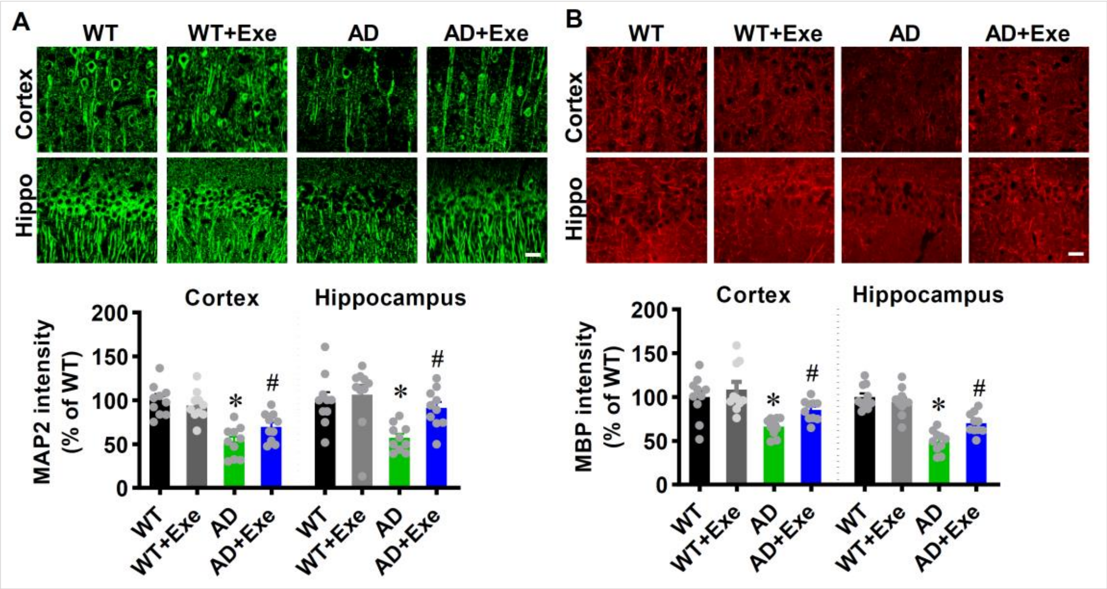

Supplementary Fig. 2. Exercise training alleviates neuronal damage in the transgenic APP/PS1 mice. (A) Representative confocal microscopy images of MAP2. The MAP2 intensity was analyzed as a percentage of WT. **(B)** Representative confocal microscopy images of MBP. The MBP intensity was analyzed as a percentage of WT. The scale bar represents 10 μm. T Data represent mean ± SEM, n = 10 slices from 6 animals. $^{*}P < 0.05$ vs. WT group, $^{\#}P < 0.05$ vs. AD group.

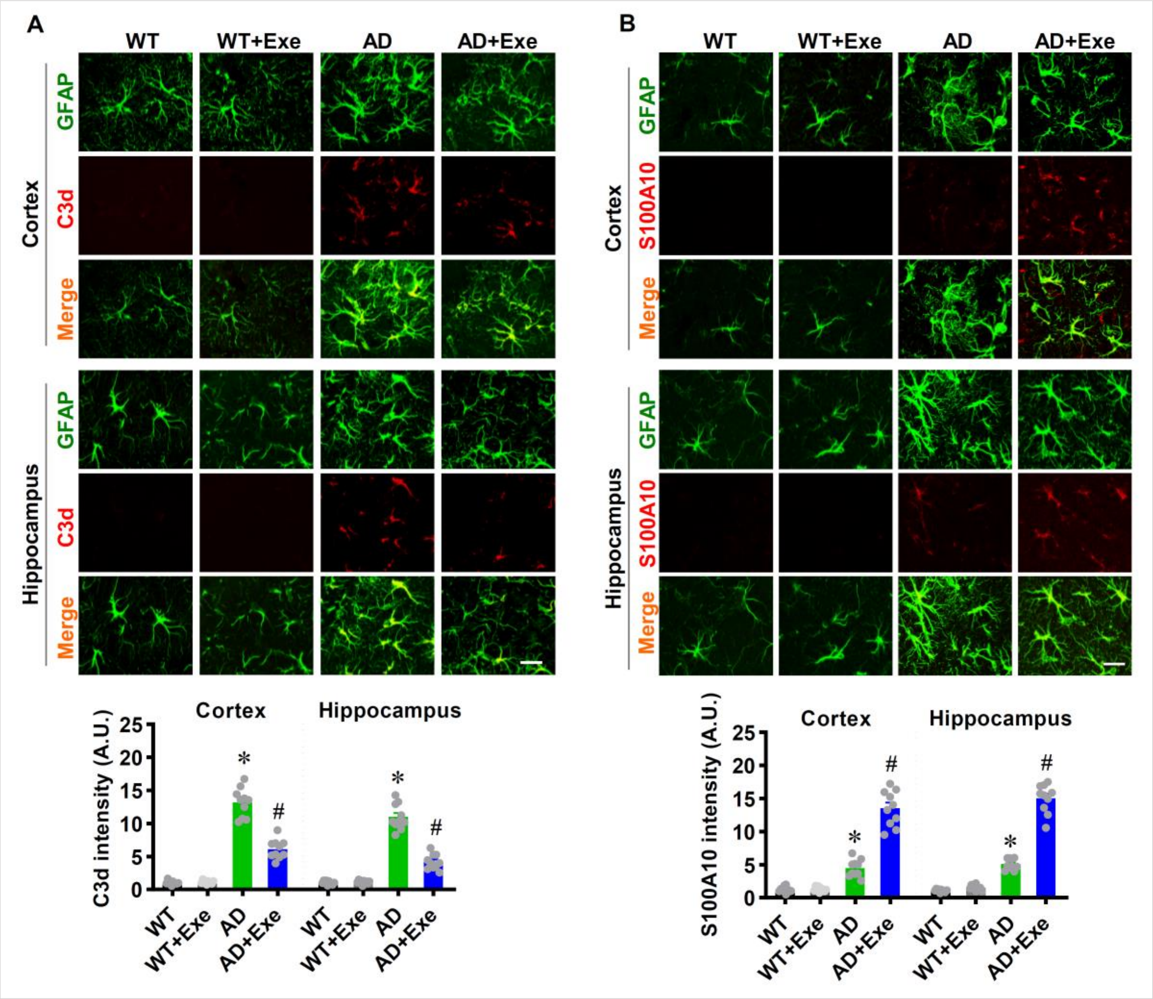

Supplementary Fig. 3. Exercise training induces astrocyte phenotype polarization toward the A2 phenotype in the transgenic APP/PS1 mice. (A) Representative confocal microscopy shows co-staining of C3d (red, A1 marker) and GFAP (green). C3d intensities in the cortex and hippocampus were measured and analyzed. **(B)** Representative confocal microscopy shows co-staining of S100A10 (red, A2 marker) and GFAP (green). S100A10 intensities in the cortex and hippocampus were measured. The scale bar represents 10 μm. A.U. indicates arbitrary units. Data represent mean ± SEM, n = 10 slices from 5 animals. $^{*}P < 0.05$ vs. WT group, $^{\#}P < 0.05$ vs. AD group.

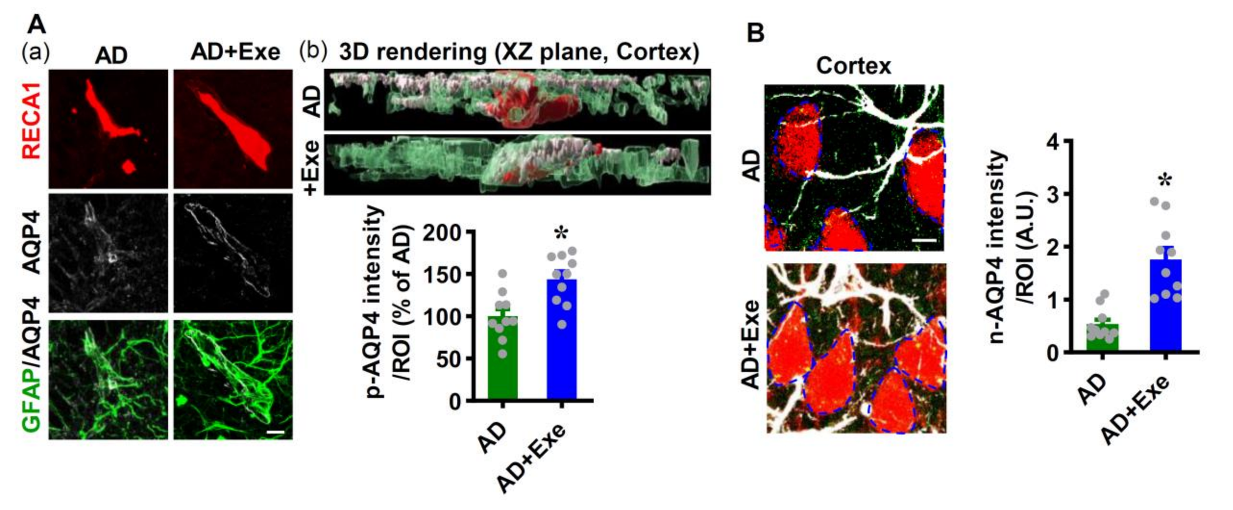

Supplementary Fig. 4. Exercise training preserves astrocyte p-AQP4 and n-AQP4 polarity in the cortex of the transgenic APP/PS1 mice. (A) Confocal immunofluorescence triple staining for RECA1 (red), AQP4 (white), and GFAP (green) (a). XZ-plane view of the 3D rendering images (b). p-AQP4 intensities within the ROI were analyzed. The scale bar represents 10 μm. **(B)** Confocal immunofluorescence triple staining for NeuN (red), AQP4 (green), and GFAP (white). n-AQP4 intensities within ROI (marked by blue dashed lines) were analyzed. A.U. indicates arbitrary units; ROI indicates the region of interest. The scale bar represents 5 μm. Data represent mean ± SEM, n = 10 slices from 5 animals. $^{*}P < 0.05$ vs. AD group.

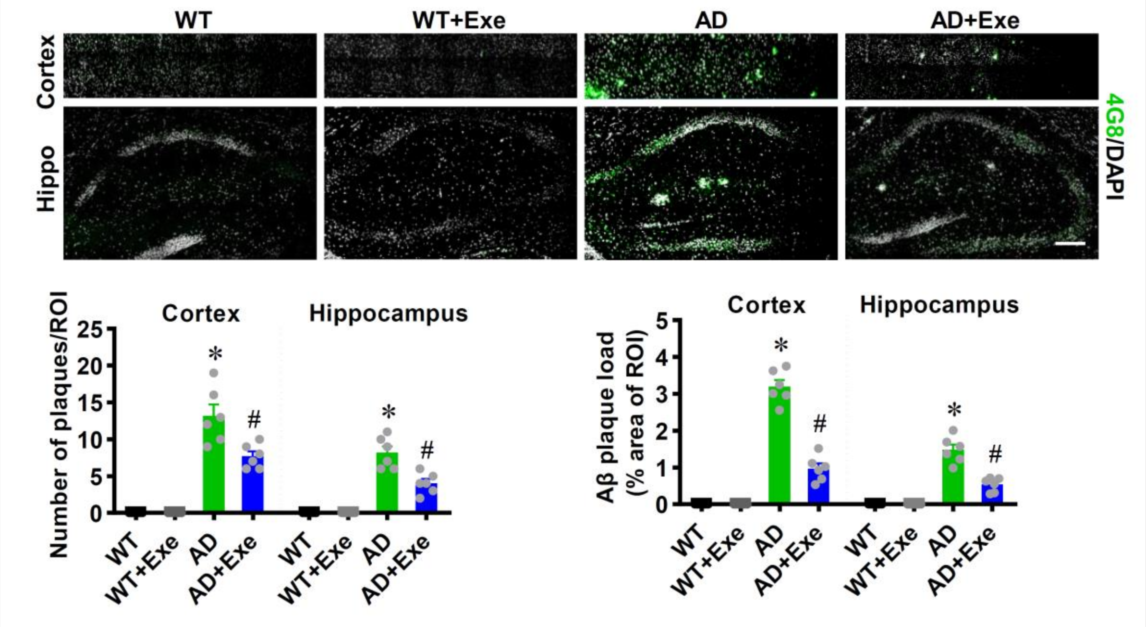

Supplementary Fig. 5. Exercise training attenuates amyloid load in the cortex and hippocampus in the transgenic APP/PS1 mice. Representative immunofluorescence images of Aβ (4G8, green) and DAPI (white) in the cortex and hippocampus. The number of amyloid plaques and Aβ plaque load in the cortex and hippocampus were quantified and analyzed. The scale bar represents 200 μm. Data represent mean ± SEM, n = 6. $^{*}P < 0.05$ vs. WT group, $^{\#}P < 0.05$ vs. AD group.

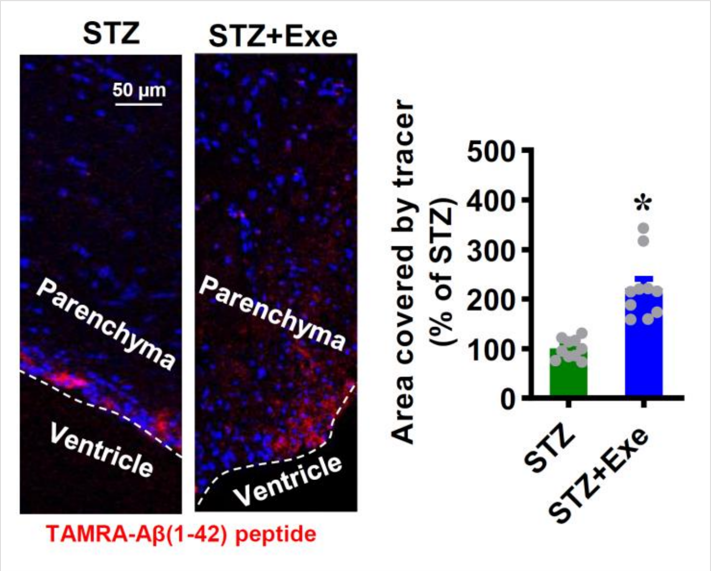

Supplementary Fig. 6. Exercise promotes glymphatic transport. A CSF tracer diffusion experiment to measure glymphatic transport. 8-μl fluorescently labeled Aβ (1 μg/μl) was bilaterally i.c.v injected following 8-week treadmill training in an STZ-induced AD rat model. Representative immunofluorescence images of TAMRA-Aβ (1-42) peptide (red) were shown, and the area covered by the tracer was analyzed. The scale bar represents 50 μm. Data represent mean ± SEM, n = 10 slices from 5 animals. $^{*}P < 0.05$ vs. STZ group.

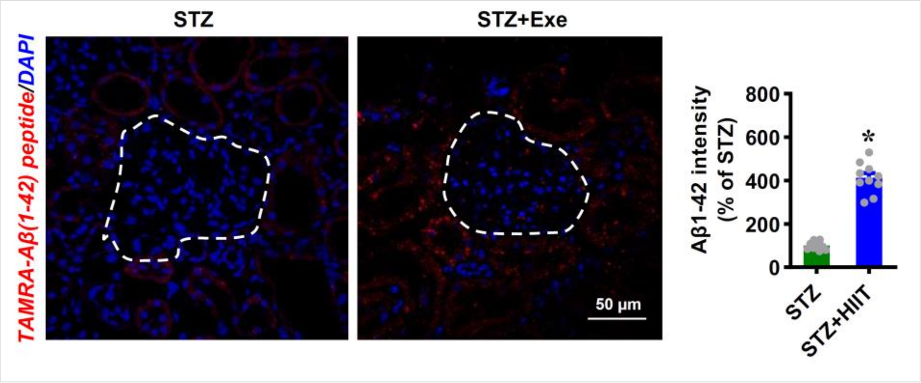

Supplementary Fig. 7. Exercise promotes the clearance of i.c.v injected Aβ from the brain to the kidney. Representative confocal microscopy of the intracerebroventricular injected TAMRA-Aβ(1-42) peptide (red) in the kidney. The intensity of the fluorescently labeled Aβ in the kidney was analyzed. The white dashed lines show positions of glomerular. The scale bar represents 50 μm. Data represent mean ± SEM, n = 10 slices from 5 animals. $^{*}P < 0.05$ vs. STZ group.
